# Dissecting the molecular diversity and commonality of bovine and human treponemes identifies key survival and adhesion mechanisms

**DOI:** 10.1371/journal.ppat.1009464

**Published:** 2021-03-29

**Authors:** Gareth J. Staton, Simon R. Clegg, Stuart Ainsworth, Stuart Armstrong, Stuart D. Carter, Alan D. Radford, Alistair Darby, Jonathan Wastling, Neil Hall, Nicholas J. Evans

**Affiliations:** 1 Department of Infection Biology & Microbiomes, Institute of Infection, Veterinary and Ecological Sciences, University of Liverpool, Leahurst Campus, Neston, United Kingdom; 2 School of Life Sciences, College of Science, University of Lincoln, Brayford Pool Campus, Lincoln, United Kingdom; 3 Faculty of Natural Sciences, Keele University, Keele, Staffordshire, United Kingdom; 4 Earlham Institute, Norwich Research Park, Norwich, United Kingdom; 5 School of Biological Sciences, University of East Anglia, Norwich, United Kingdom; 6 Department of Biological Sciences, King Abdulaziz University (KAU), Jeddah, Saudi Arabia; Stanford University School of Medicine, UNITED STATES

## Abstract

Here, we report the first complete genomes of three cultivable treponeme species from bovine digital dermatitis (DD) skin lesions, two comparative human treponemes, considered indistinguishable from bovine DD species, and a bovine gastrointestinal (GI) treponeme isolate. Key genomic differences between bovine and human treponemes implicate microbial mechanisms that enhance knowledge of how DD, a severe disease of ruminants, has emerged into a prolific, worldwide disease. Bovine DD treponemes have additional oxidative stress genes compared to nearest human-isolated relatives, suggesting better oxidative stress tolerance, and potentially explaining how bovine strains can colonize skin surfaces. Comparison of both bovine DD and GI treponemes as well as bovine pathogenic and human non-pathogenic saprophyte *Treponema phagedenis* strains indicates genes encoding a five-enzyme biosynthetic pathway for production of 2,3-diacetamido-2,3-dideoxy-d-mannuronic acid, a rare di-*N*-acetylated mannuronic acid sugar, as important for pathogenesis. Bovine *T*. *phagedenis* strains further differed from human strains by having unique genetic clusters including components of a type IV secretion system and a phosphate utilisation system including *phoU*, a gene associated with osmotic stress survival. Proteomic analyses confirmed bovine derived *T*. *phagedenis* exhibits expression of PhoU but not the putative secretion system, whilst the novel mannuronic acid pathway was expressed in near entirety across the DD treponemes. Analysis of osmotic stress response in water identified a difference between bovine and human *T*. *phagedenis* with bovine strains exhibiting enhanced survival. This novel mechanism could enable a selective advantage, allowing environmental persistence and transmission of bovine *T*. *phagedenis*. Finally, we investigated putative outer membrane protein (OMP) ortholog families across the DD treponemes and identified several families as multi-specific adhesins capable of binding extra cellular matrix (ECM) components. One bovine pathogen specific adhesin ortholog family showed considerable serodiagnostic potential with the *Treponema medium* representative demonstrating considerable disease specificity (91.6%). This work has shed light on treponeme host adaptation and has identified candidate molecules for future diagnostics, vaccination and therapeutic intervention.

## Author summary

Bovine digital dermatitis (DD) is a severe infectious disease causing cattle lameness, which is now endemic in many countries across the world. This lameness results from inflamed lesions between the heel bulbs and is very painful resulting in poor animal welfare and substantially reduced production. There remains no single cure for DD and whilst topical antibiotic treatment enables some healing, lesions frequently reappear. Current evidence implicates bacteria known as *Treponema* in the pathogenesis of DD. Here we characterise the genomes of several bovine DD treponeme species as well as related bacteria from humans and the bovine gastrointestinal tract. Comparative analyses demonstrate that production of a novel mannuronic acid sugar is a key feature of bovine pathogens and several survival mechanisms were identified which likely enable the bovine pathogens to inhabit the skin surface and be transmitted within the farm environment. Studies investigating putative outer membrane proteins which are potential vaccine candidates identified that the majority have a role in host attachment, with one family of proteins exhibiting particular promise as serodiagnostic antigens. This increased understanding of the considered causal pathogens of bovine DD, together with the genomic and proteomic resources produced by this study should underpin future diagnostic, vaccination and therapeutics studies to combat this severe disease of ruminants.

## Introduction

The *Treponema* were first described in detail by Schaudinn and Hoffman in 1905 during the discovery of the agent of syphilis [1] and are a continually expanding genus of bacteria with diverse roles in a variety of niches. These spiral microorganisms are generally considered anaerobic, have a fastidious nature making their study difficult, and have been frequently reported within human and animal oral, genital and rectal areas as well as the broader gastrointestinal (GI) tract [2–4]. This important genus has a complicated relationship with disease aetiology ranging from single taxa responsible for human and rabbit syphilis, *Treponema pallidum* and *Treponema paraluiscuniculi* [5], the multiple phylogroups considered to act synergistically as a polytreponemal aetiology in bovine digital dermatitis (BDD) [6–8] and important species such as *Treponema medium* and *Treponema denticola* [9,10], which are implicated in the more broadly polymicrobial aetiologies of human and canine periodontal disease [11–13]. The presence of treponemes existing as commensals within animals has been reported with *Treponema bryantii* and *Treponema ruminis* isolated from the rumen of cows [14,15], whilst *Treponema rectale* has been isolated from the bovine rectum [16] and further novel *Treponema* have been derived from pig faecal material [17,18]. In humans, several treponemes were coincidently isolated during the original pursuit of the syphilis agent, with *Treponema phagedenis* particularly well studied as a comparator of syphilis and considered saprophytic and non-pathogenic [19]. Due to the fastidiousness of *Treponema* spp., many taxa remain uncultured or poorly characterised, typically only being described by their 16S rRNA gene sequence identity.

Whilst other less fastidious bacteria have been voraciously genome sequenced, complete genome sequences and comparative analyses of treponemes have been much slower due to the arduous nature of treponeme culture and isolation. Despite microbiome studies continuing to emphasise the ubiquitous nature of treponemes within human and animal samples [20–23] there are still only limited numbers of isolates available globally. During the 20^th^ century, a large number of researchers focused on the human and non-human primate treponematoses which culminated in the genome sequencing of the agents of human syphilis and yaws. These treponemes exhibited a small bacterial genome with respective genome sequences differing less than 0.2% [24,25] and later comparative genomic analyses with rabbit syphilis treponemes identified that loss of human infectivity is attributable to genome decay [26]. Another focus has been on the periodontal treponemes with the *T*. *denticola* genome completed and broad comparisons drawn with *T*. *pallidum* identifying a substantial difference in genome size due to lateral gene transfer, genome degradation and lineage-specific expansions [9]. At the turn of this century, an important and highly contagious global cattle disease, BDD, was recognised as having spread around the world, gaining endemic status in USA and Europe [27–29]. This infectious disease of cattle feet, which causes severe lameness has important animal welfare and economic implications and raises substantial antibiotic stewardship issues. Previously, we isolated and characterised BDD treponemes using phylogenetic and polyphasic phenotyping [30,31] and reported, together with others, substantial associations between three specific phylogroups, *T*. *medium*-like, *T*. *phagedenis*-like and *Treponema pedis* (formerly *T*. *denticola*-like) and BDD [7,8,31,32]. A German BDD study using FISH identified these groups as most important for aetiology [8], with these key phylogroups now also implicated globally as key for disease aetiology [33,34]. We have additionally isolated novel bovine GI tract *Treponema* as BDD treponeme comparators, identifying them as characteristically different [35]. The generation and comparative analysis of these bovine disease causing treponemes against other relevant human and animal treponemes should further enable understanding of this complex genus of bacteria and the roles they play in diseases.

Here, we have sequenced and present the complete genomes of six treponemal strains representative of four different spirochete species of human and bovine origin. The comparative genomic analyses and subsequent phenotypying including proteomics undertaken here identifies key insights into the biology of these pathogens.

## Results

### Genome sequencing bovine and human *Treponema* representatives identifies a reduced coding density in bovine pathogenic strains and diversity in homopolymeric tract distributions

To better understand pathogenesis across the *Treponema* genus, we produced complete genomes for key pathogenic and commensal treponemes isolated from both bovine and human origins. The genomes sequenced included a representative from each of three unique phylogroups of treponemes considered the major aetiological agents of BDD including *Treponema medium* strain T19, *Treponema phagedenis* strain T320A and *Treponema pedis* strain T3552B^T^ [7,8,31, 33,34]. To enable comparisons relating to pathogenesis and host adaptation, we also included the human oral pathogen *T*. *medium* strain ATCC 700293^T^ [10], the considered saprophytic, non-pathogenic human derived *Treponema phagedenis* biotype Reiter [19], as well as a bovine commensal treponeme isolated from a dairy cow rumen, *Treponema ruminis* Ru1^T^ [14]. General features of the newly sequenced genomes are listed in Table 1. The genomes were all 2.72–3.12Mb in size, larger than the rabbit and human syphilis treponeme genomes (~1Mb) and of a similar size to *T*. *denticola* ATCC 35405 (2.84Mb). Bovine pathogenic treponemes were each larger than their identified closest genetic relative derived from human tissues [31] with bovine *T*. *medium* T19 larger than the human pathogenic *T*. *medium* ATCC 700293^T^ (164.6kb), bovine *T*. *phagedenis* T320A larger than the human saprophytic strain Reiter (239.6kb) and *T*. *pedis* strains T3552B^T^ larger than human *T*. *denticola* ATCC 35405 (46.2kb). Furthermore, there were more pseudogenes in the bovine treponeme genomes than human representatives (Table 1). There was a substantial number of pseudogenes for bovine *T*. *pedis* T3552B^T^, more so than for both porcine *T*. *pedis* A4 and *T*. *denticola* ATCC 35405, with a number of these resulting from rearrangement hotspot (RHS) genes and homopolymeric tracts (HTs).

**Table 1:** General features of human and bovine representatives of *T*. *medium* and *T*. *phagedenis*, bovine *T*. *pedis*, and bovine *T*. *ruminis* compared (six new complete genomes) with human *T*. *denticola*, porcine *T*. *pedis* and human and rabbit syphilis treponemes.

Category^a^	*Treponema medium* ATCC 700293^T^	*Treponema medium* T19 DSM 18689	*Treponema phagedenis* Reiter	*Treponema phagedenis* T320A DSM 18690	*Treponema pedis* T3552B^T^ DSM 18691	*Treponema pedis* A4	*Treponema ruminis* DSM 103462^T^	*Treponema denticola* ATCC 35405	*Treponema pallidum subsp*. *pallidum Nichols*	*T*. *paraluiscuniculi Cuniculi A*
Host	human	bovine	human	bovine	Bovine	Porcine	bovine	human	human	rabbit
Genbank Accession No^b^	**CP031393**	**CP027017**	**CP031394**	**CP027018**	**CP045760**	NC_022099.1	**CP031518**	NC_002967.9	NC_021490.2	NC_015714.1
Genome size (bp)	2,721,998	2,886,632	2,880,698	3,120,315	2,889,390	2,889,325	2,895,582	2,843,201	1,139,633	1,133,390
G + C content (%)	44.3	44.3	40.2	39.9	36.9	36.9	44.6	37.9	52.8	52.7
Genes	2409	2564	2596	2858	2694	2645	2525	2838	1065	1062
Protein coding genes	2352	2504	2545	2804	2642	2593	2466	2767	1011	1008
Pseudogenes	44	78	60	94	196	120	17	19	7	32
tRNA	48	51	45	45	43	43	49	44	45	45
16S/23S/5S	2/2/2	2/2/2	2/2/2	2/2/2	2/2/2	2/2/2	2/2/2	2/2/2	2/2/2	2/2/2
Coding density (%)	86.2	84.6	81.3	81.0	80.7	85.1	92.4	91.7	93.2	90.7
Putative Homo-polymeric tracts^c^	1320 T 1343 A 30 G 19 C	1403 T 1408 A 33 G 20 C	2854 T 2686 A 16 G 9C	2979 T 3015 A 17 G 23 C	3505 T 3230 A 40 G 38 C	3499 T 3241 A 39 G 31 C	1274 T 1253 A 9 G 6 C	2997 T 3031 A 8 G 18 C	250 T 311 A 53 G 47 C	248 T 315 A 48 G 50 C

^a^All according to the latest genome annotations at the time of writing.

^**b**^Accession number of each genome analysed with genome sequences submitted as part of this study identified in bold.

^c^ HTs are seven or more identical nucleotides in a row [100].

Coding density was much lower for the BDD treponemes (80.7–86.2%) than for *T*. *denticola* ATCC 35405, the two syphilis treponemes and the *T*. *ruminis* Ru1^T^ genome (90.7–93.2%). The GC content of the bovine and human treponemes (37.9–44.6%) was considerably lower than both the syphilis genomes (52.7–52.8%). The number of putative HTs per genome demonstrated a wide variation across species. The largest number of G and C HTs were in the two syphilis treponeme genomes ([48–53] / [47–50] G/C tracts: Table 1) with only *T*. *pedis* strains having a similar number ([39–40] / [31–38] G/C tracts). The syphilis genomes comparatively exhibit a five or ten fold decrease in A and T HTs compared with the cultivable treponemes which coincides with the higher GC content of the syphilis treponeme genomes. The considered GI commensal, bovine *T*. *ruminis* Ru1^T^, exhibited the least HTs. No plasmids were identified in any of the genomes.

### Surveying recognised Gram-negative bacterial survival- and virulence- genes identifies bovine pathogenic strains have more oxidative stress genes than nearest human relatives

Discerning mechanisms of host adaptation or differentiating pathogens from commensals typically identifies novel virulence or survival mechanisms. Here, we surveyed recognised Gram-negative bacterial genes involved in mechanisms of bacterial survival or virulence, within the different select treponemes (Table 1) with results shown in Table 2. We screened for the presence of eukaryotic-like domains (ELD) which may act as host cell effectors and tend to be components of secretion systems. Using a relevant prediction program (EffectiveELD), no complete secretion systems could be identified in any of the treponemes. In terms of eukaryotic-like domains (ELDs) there was a clear numerical increase when comparing bovine and human representatives from the same species for both *T*. *medium* and *T*. *phagedenis*. This expansion in ELDs within the bovine pathogenic strain of *T*. *phagedenis* included presence of dynamin family domains, DNA methylase and winged helix-turn helix domains whilst bovine *T*. *medium* included polymer-forming cytoskeletal and ribosomal domains (S1 File). BspA type leucine rich repeat region domains were a common ELD feature of all treponeme genomes surveyed except *T*. *ruminis* Ru1^T^, porcine *T*. *pedis* A4 and the two syphilis treponemes. The commensal *T*. *ruminis* Ru1^T^ had the fewest predicted ELDs whilst both porcine *T*. *pedis* A4 and human *T*. *denticola* ATCC 35405 had more than bovine *T*. *pedis* T3552B^T^. A variety of bacteria (including *T*. *denticola* ATCC 35405) contain rearrangement hotspot (RHS) proteins containing YD-dipeptide repeats (RHS/YD repeats) which together can act as toxins inhibiting/damaging nearby bacterial and host cells. Here, RHS/YD repeats were only present in the *T*. *pedis* genomes in addition to the periodontal pathogen *T*. *denticola* ATCC 35405. In terms of toxins responsible for host damage, both *T*. *medium* genomes contain Tetanolysin O, with bovine *T*. *medium* T19 having an additional paralog of this gene. Gene encoding hemolysins are present in all the treponemes, including the non-pathogenic human *T*. *phagedenis* Reiter saprophyte but not the bovine GI treponeme commensal *T*. *ruminis* Ru1^T^.

**Table 2:** Distribution of unique and shared Gram-negative bacterial survival and virulence associated genes across bovine and human treponemes.

	*Treponema medium* ATCC 700293^T^	*Treponema medium* T19 DSM 18689	*Treponema phagedenis* Reiter	*Treponema phagedenis* T320A DSM 18690	*Treponema pedis* T3552B^T^ DSM 18691	*Treponema pedis* A4	*Treponema ruminis* DSM 103462^T^	*Treponema denticola* ATCC 35405	*Treponema pallidum subsp*. *pallidum Nichols*	*T*. *paraluiscuniculi* *Cuniculi A*
Host	human	bovine	Human	Bovine	Bovine	porcine	bovine	human	human	rabbit
Hemolysins^**1**^	3	3	3	3	3	3	0	4	1	1
Toxins^**1**^	Tetanolysin O (1)	Tetanolysin O (2)	0	0	0	0	0	0	0	0
Eukaryotic-like Domains (ELDs)/Proteins containing ELDs ^**2**^	18/42	41/72	22/45	27/112	23/55	23/81	18/46	27/114	6/8	5/6
Complete secretion systems^**2**^	0	0	0	0	0	0	0	0	0	0
RHS/YD repeats^**1**^	0	0	0	0	47	45	0	4	0	0
Oxidative stress related^3^	42	46	43	44	49	49	42	40	24	24
Toxin:Antitoxin systems^**1**^	VapC (8) HicA (3) PIN HicB RelE/ParE (6) PemK/MazF BrnT (8) RelE (2) RelB/DinJ (5) RelB/StbE PhD/YefM (4) Txe/YoeB (2) AbiEii/AbiGii	VapC (8) HicA (3) PIN HicB RelE/ParE (7) PemK/MazF BrnT (10) RelE (2) RelB/DinJ (4) RelB/StbE PhD/YefM (4) Txe/YoeB (2) AbiEii/AbiGii	HicA Txe/YoeB	HicA Txe/YoeB	RelE/ParE (2) RelB/DinJ (3) Txe/YoeB (3) YafQ VapC (2) PhD/YefM	RelE/ParE (2) RelB/DinJ (3) Txe/YoeB (3) YafQ VapC (2) PhD/YefM	RelE/ParE (2) VapC (3) HipA HicB PIN PhD/YefM	VapC	0	0

^1^Based on genome annotation and Mauve [80] alignment with ortholog 50% sequence identity.

^2^As determined using a relevant bioinformatics package, EffectiveELD [79].

^3^As determined according to S1 Table.

**Table 3:** Distribution of novel orthologs differentiating bovine pathogenic and commensal treponemes.

	*Treponema medium* ATCC 700293^T^	*Treponema medium* T19 DSM 18689	*Treponema phagedenis* Reiter	*Treponema phagedenis* T320A DSM 18690	*Treponema pedis* T3552B^T^ DSM 18691	*Treponema pedis* A4	*Treponema ruminis* DSM 103462^T^	*Treponema denticola* ATCC 35405	*Treponema pallidum subsp*. *pallidum Nichols*	*T*. *paraluiscuniculi* *Cuniculi A*
Host	human	bovine	Human	Bovine	Bovine	porcine	bovine	human	human	rabbit
pyruvate kinase (PK) (C5O78_13165) *(CYT)* ^*1*^	+ *(E)*	+ *(E)*	+ *(E)*	+ *(E)*	+ *(E)*	+ *(E)*	-	+	-	-
UDP-N-acetyl-D-glucosamine dehydrogenase (WbpA/WbpO) (C5O78_00350) *(CYT)*	+ *(E)*	+ *(E)*	-	+ *(E)*	+ *(E)*	+ *(E)*	-	-	-	-
UDP-N-acetyl-2-amino-2-deoxyglucuronate dehydrogenase (WbpB/WlbA) (C5O78_00440) *(CYT)*	+ *(E)*	+ *(E)*	-	+ *(E)*	+ *(E)*	+ *(E)*	-	-	-	-
UDP-2-acetamido-2-deoxy-ribo-hexuluronate aminotransferase (WbpE/WlbC) (C5O78_00420) *(CYT)*	+ *(E)*	+ *(E)*	-	+ *(E)*	+ *(E)*	+ *(E)*	-	-	-	-
UDP-2-acetamido-3-amino-2,3-dideoxy-glucuronate N-acetyltransferase (WbpD/WlbB) (C5O78_00405) *(CYT)*	+ *(E)*	+ *(E)*	-	+ *(E)*	+ *(E)*	+	-	-	-	-
UDP-GlcNAc3NAcA epimerase (WbpI /WlbD) (C5O78_10860) *(CYT)*	+ *(E)*	+ *(E)*	-	+ *(E)*	+ *(E)*	+ *(E)*	-	-	-	-
magnesium transporter (MgtE) (C5O78_12755) *(IM)*	+ *(E)*	+ *(E)*	+ *(E)*	+ *(E)*	+ *(E)*	+ *(E)*	-	+	+	+
PPIA; peptidyl-prolyl cis-trans isomerase A (cyclophilin A) (C5O78_00570) *(CYT)*	+ *(E)*	+ *(E)*	+ *(E)*	+ *(E)*	+ *(E)*	+ *(E)*	-	+	-	-
chaperonin GroEL (groEL) (C5O78_01775) *(CYT)*	+ *(E)*	+ *(E)*	+ *(E)*	+ *(E)*	+ *(E)*	+ *(E)*	-	+	+	+

^**1**.^Predicted location of proteins are listed after each molecule in parentheses. IM = Inner membrane, CYT = Cytoplasm, none localised to outer membrane. Expression of corresponding protein identified using proteomics is denoted by *E* in parentheses. Identification of expression by the respective treponemal species (when grown in routine culture) as determined by Nano LC MS/MS analysis. All putative OMPs detected in a minimum of two peptide identifications.

A range of bacterial toxin:antitoxin systems were identified within the *T*. *medium* genomes with *T*. *pedis* strains and *T*. *ruminis* Ru1^T^ also having a considerable number, contrasting a lower number in other genomes and absence in the syphilis treponemes (Table 2).

There were a substantial number of oxidative stress related genes identified across the treponemes, including many NADP and FAD oxidoreductases (Tables 2 and S1). Whilst the syphilis treponemes possess 24 aerotolerance-associated genes, the other treponemes have twice this number of oxidative genes whilst having genomes three times larger. For *T*. *medium* T19 and *T*. *phagedenis* T320A, the bovine strains had either four or one more oxidative stress genes respectively than the human strains. Both animal *T*. *pedis* strains had nine more oxidative stress genes than human *T*. *denticola* ATCC 35405.

### Distribution of recognised treponemal virulence associated genes across bovine and human treponemes frequently fails to discern pathogenic ability or host adaptation

A comparative analysis of recognised virulence factors of *T*. *denticola* and *T*. *pallidum* was used to investigate putative virulence factor distribution across the treponemes (S2 Table). Some virulence factors appear to be present across all treponemes or differentiate specific treponeme groups such as putative pathogens. The commensal from the bovine GI tract, *T*. *ruminis* Ru1^T^, lacks the majority of virulence genes. All genomes except *T*. *ruminis* have some representation of the reported Tmp ABC orthologs. Based on orthology criteria employed here, pallilysin (Tp0751) is restricted to the two syphilis treponemes whilst the polycistronic partner Tp0750 is present in all treponemes except for *T*. *ruminis* Ru1^T^. The bovine commensal spirochete *T*. *ruminis* Ru1^T^ was differentiated from all others surveyed by the absence of three genes (*tlyC*, *hlyIII* and *cfpA*) and, when excluding syphilis treponemes, could be differentiated by the absence of a further two genes (*tp0750*, *hly*). The human *T*. *phagedenis* Reiter saprophyte could not be differentiated from pathogenic treponemes by these recognised virulence factors nor could human and bovine derived *T*. *medium* be discriminated on these criteria.

### Comparative genomics of commensal and pathogenic cultivable treponemes implicates a five-enzyme biosynthetic pathway for 2,3-diacetamido-2,3-dideoxy-d-mannuronic acid as important to pathogenesis

**Fig 1:**
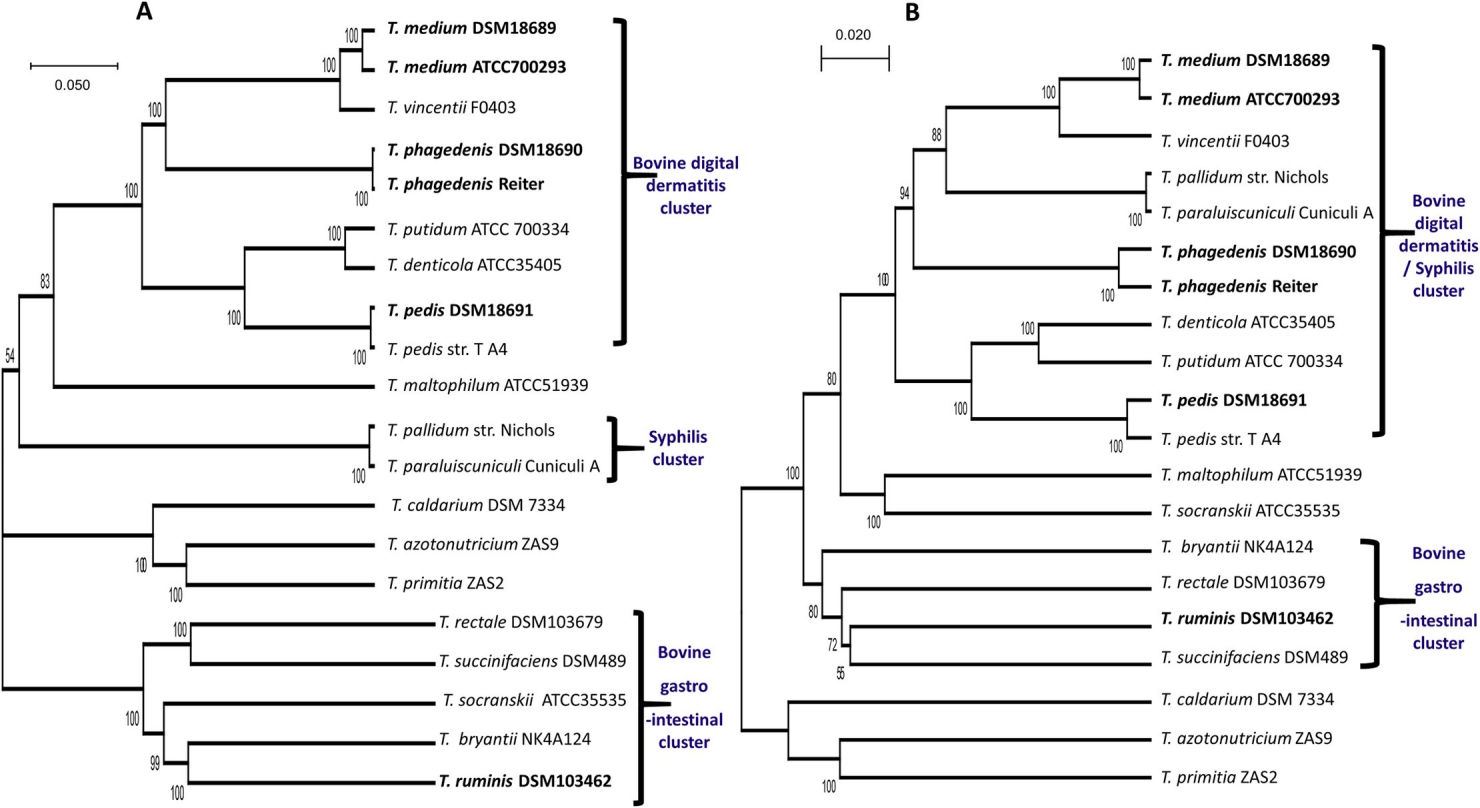
Phylogenetic trees of core and pan genomes from relevant treponemes. A core-genome phylogenetic tree was constructed using protein sequences from 20 random orthologous gene clusters (encoded by ileS, valS, topA, fusA, fusA-2, tpiA, dnaK, lepA, atpA, fliI, flgE, eno, atpB, clpX, metK, dnaJ, ruvB, and uncharacterised genes corresponding to *T*. *denticola* ATCC35405 genome loci TDE0714 and TDE1969, with a total concatenated size of 36.12kb) aligned in MUSCLE [87], and subjected to the unweighted pair group method with arithmetic mean (UPGMA) algorithm and the Jones-Taylor-Thornton (JTT) model with 10,000 bootstrap replicates [88] (**A**). A pan-genome phylogenetic tree was reconstructed using UPGMA algorithm and the Jukes-Cantor model with 10,000 bootstrap replicates [88] using a binary presence/absence gene pan matrix produced from BPGA representative of 23,260 genes [75] (**B**).

Comparative genomics allows for *de novo* discovery of key differences and similarities between bacteria including mechanisms of host adaptation, pathogenesis and survival. Here, construction of a phylogenetic tree of relevant available treponeme genome sequences based on the core genome resulted in the bovine DD, bovine GI and syphilis treponemes each dividing into their own phylogenetic clusters (Fig 1A). Contrastingly, a pan genome phylogenetic tree of the same taxa grouped the bovine DD treponemes together with the syphilis treponemes (Fig 1B). Comparing a number of ribosomal genes from the treponemes, in a riboMLST phylogenetic tree [36], produced the same groupings as the core genome phylogenetic analysis (S1 Fig).

**Fig 2:**
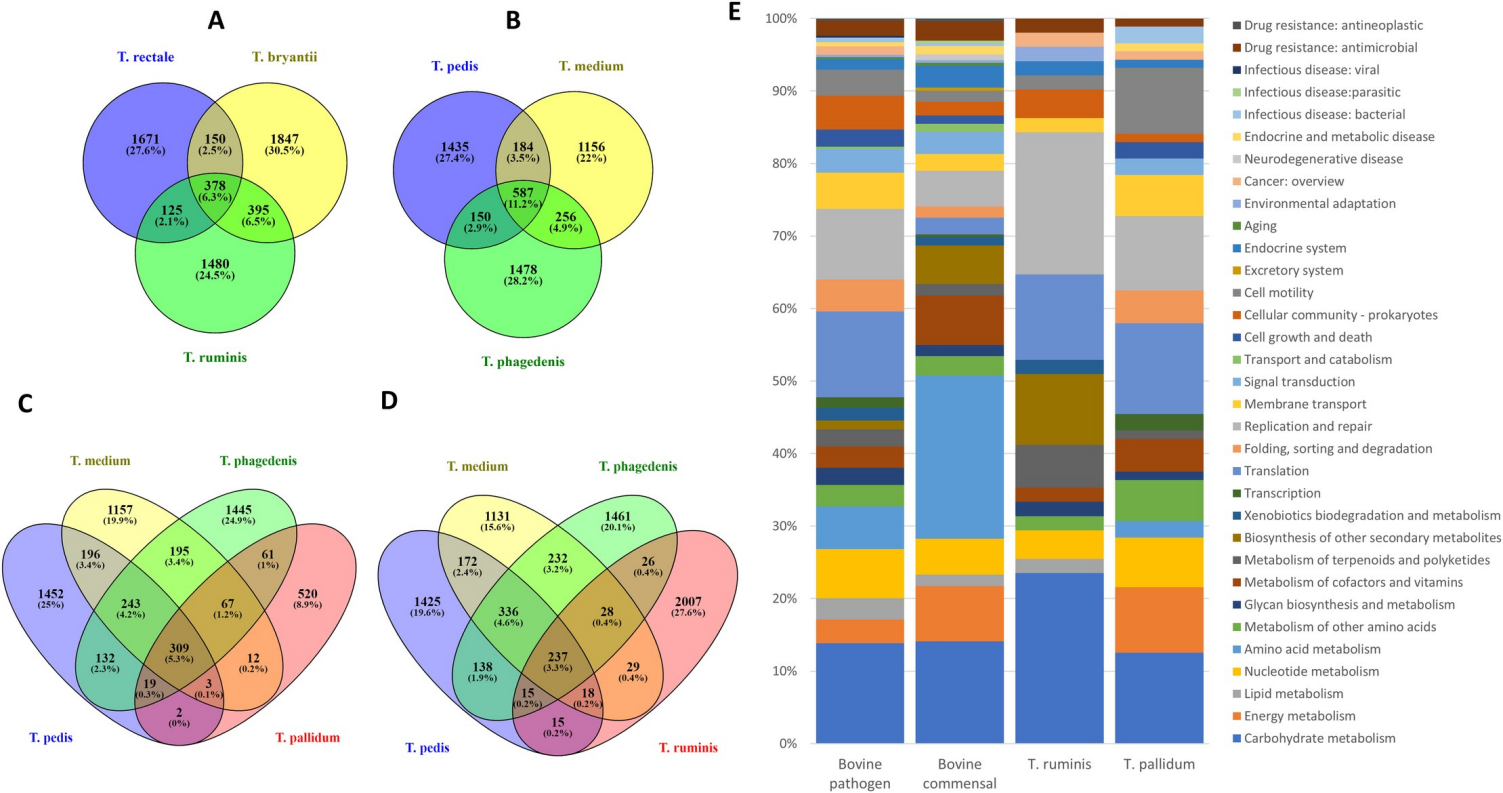
Core, accessory and unique genes shared between bovine digital dermatitis and bovine gastrointestinal treponemes or shared between bovine digital dermatitis treponemes with the pathogen *Treponema pallidum* or the commensal gastrointestinal *Treponema ruminis*. Venn diagram representation of the core, accessory and unique genes shared between bovine GI treponemes (**A**) and bovine digital dermatitis treponemes (**B**) or shared between bovine digital dermatitis treponemes with the pathogen *Treponema pallidum* (**C**) or the commensal GI *Treponema ruminis* (**D**). Numbers of shared gene families and/or unique genes are listed with the percentage of the total pan-genome this represents (for each comparison) listed in parentheses. **E:** After the core treponeme genome shared between the BDD treponemes and bovine GI treponemes were removed, functional annotation was carried out on the shared unique genes for the BDD treponemes and the bovine GI treponemes. Only 189 entries (79.4%) annotated of the 238 uniquely shared genes across the bovine GI treponemes could be annotated (**Bovine Commensal**), only 325 entries (74.2%) annotated of 438 uniquely shared genes across the BDD treponemes could be annotated (**Bovine Pathogen**). After the core treponeme genome shared between the BDD treponemes and both *T*. *pallidum* and *T*. *ruminis* were removed, functional annotation was carried out on remaining uniquely shared genes with either *T*. *pallidum* or *T*. *ruminis*. Only genes that could be assigned a KEGG category are represented in the bar graphs. Only 96 entries (75.0%) annotated of the 128 uniquely shared genes between BDD treponemes and ***T*. *pallidum*** could be annotated, only 47 entries (92.2%) annotated of 51 uniquely shared genes between BDD treponemes and ***T*. *ruminis*** could be annotated.

To investigate differences between commensal and pathogenic treponemes, the three bovine DD treponeme genomes sequenced here were compared with three commensal bovine treponemes. Bovine GI treponemes *T*. *ruminis*, *T*. *rectale* and *T*. *bryantti* shared 83.9–90.0% sequence identity when 1320 bases of the 16S rRNA gene were compared [35] and their Venn diagram of the pan/core genome analysis showed they shared 378 (6.3%) of genes (Fig 2A). In contrast, the three BDD treponemes share a higher 16S rRNA gene sequence identity of 90.3–91.1% with each other [35] and a larger number 587 (11.2%) of genes (Fig 2B). We implemented a BGPA pan genome analysis of all six genomes, which after removing a core genome of 127 orthologs shared across all six treponemes resulted in 438 bovine pathogen specific and 238 bovine commensal specific shared genes. Functional annotation of these genes demonstrated key differences between pathogens and commensals. The pathogens differed in having a larger number of genes associated with lipid and nucleotide metabolism, translation, replication and repair, membrane transport, cell community and motility (Fig 2E). This coincided with complete pathways for pyruvate oxidation to acetyl-CoA and fatty acid metabolism through beta-oxidation to produce acyl-CoA. Bovine commensals had a greater genomic potential for energy generation, amino acid metabolism, metabolism of co-factors and vitamins and biosynthesis of other secondary metabolites (Fig 2E). This coincided with complete pathways for ornithine biosynthesis from glutamate and nucleotide sugar biosynthesis of UDP-galactose. This comparison of bovine commensal and pathogenic treponemes allowed for the *de novo* identification of putative virulence factors (Tables 3 and S3). These include four peptidases, enzymes involved in carbohydrate metabolism including pyruvate kinase, transporters responsible for sugar import, an iron storage protein, flagellar biosynthesis, heavy metal transport, ABC transporters, phosphocarrier proteins, nucleotide synthesis and protein folding including GroEL and peptidyl-prolyl cis-trans isomerase A (PPIA; cyclophilin A). A number of enzymes involved in O-Antigen nucleotide sugar biosynthesis (WbpA/WbpO, WbpB/WlbA, WbpE/WlbC, WbpD/WlbB, WbpI/WlbD) were also uniquely present in the pathogens which encode a five-enzyme biosynthetic pathway for the production of 2,3-diacetamido-2,3-dideoxy-d-mannuronic acid (d-ManNAc3NAcA), a rare di-N-acetylated mannuronic acid sugar.

In further comparisons, the three BDD treponeme species shared 309 genes with *T*. *pallidum* (Fig 2C) but only 237 with *T*. *ruminis* (Fig 2D), a bovine commensal and this coincides with 16S rRNA gene sequence identities of 87.6%-90.1% and 80.0–81.1% respectively. Core genes shared between BDD treponemes and *T*. *pallidum* include genes relating to energy, nucleotide and amino acid metabolism, metabolism of co-factors/vitamins, transcription, translation, protein folding including GroEL and PPIA again, membrane transport, signal transduction, cell growth and death and cell motility (Fig 2E). Core genes shared between the BDD treponemes and *T*. *ruminis*, encoded functions included lipid metabolism, metabolism of terponoids and polyketides, biosynthesis of other secondary metabolites, xenobiotics metabolism, replication and repair, cellular community and environmental adaptation (Fig 2E).

### Bovine pathogenic *T*. *phagedenis* strains differ from non-pathogenic human strains due to unique genetic clusters encoding components of a type IV secretion system, a phosphate utilisation system and a biosynthetic pathway for 2,3-diacetamido-2,3-dideoxy-d-mannuronic acid

**Fig 3:**
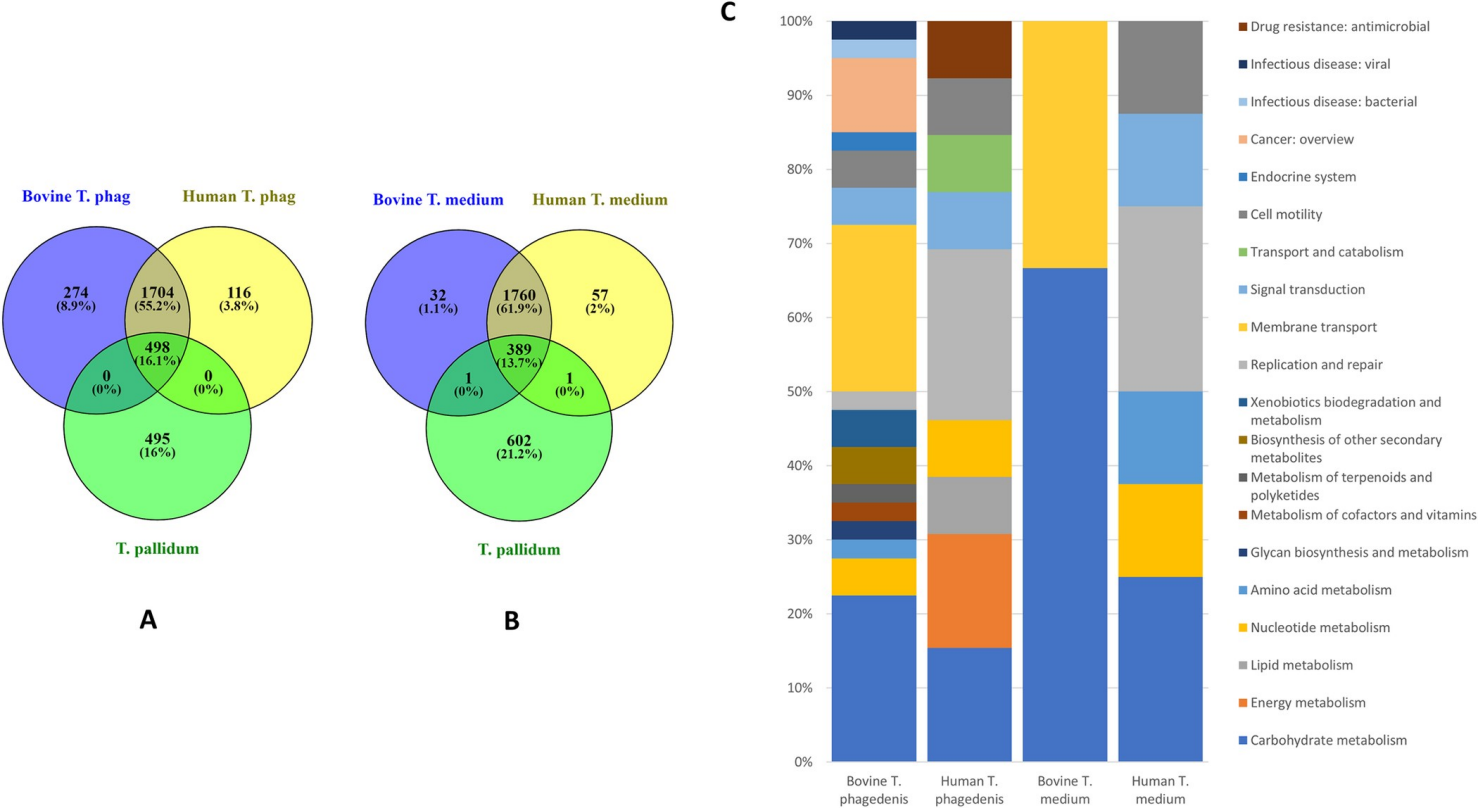
Core, accessory and unique genes shared between bovine pathogenic and human saprophyte *Treponema phagedenis* strains and *Treponema pallidum* or between bovine and human pathogenic *Treponema medium* strains and *T*. *pallidum*. Venn diagram representation of the core, accessory and unique genes shared between bovine pathogenic and human saprophyte *T*. *phagedenis* strains and *T*. *pallidum* **(A)**: Core, accessory and unique genes shared between bovine and human pathogenic *T*. *medium* strains and *T*. *pallidum* **(B)**. Numbers of shared gene families and/or unique genes are listed with the percentage of the total pan-genome this represents (for each comparison) listed in parentheses. **C:** Functional classification of genes unique to bovine and human *T*. *phagedenis* or bovine and human *T*. *medium*. Only genes that could be assigned a KEGG category are represented in the bar graphs. Only 27 (23.3%) of the 116 unique genes from human *T*. *phagedenis* could be annotated, only 68 entries (24.8%) of the 274 unique bovine *T*. *phagedenis* genes could be annotated. Only 16 (28.1%) of the 57 unique genes from human *T*. *medium* could be annotated, only 4 entries (12.5%) of the 32 unique bovine *T*. *medium* genes could be annotated.

Previous comparisons of bovine and human *T*. *phagedenis* have not identified substantial evidence for genotypic or phenotypic differences [31,37]. Here, we dissected genomic variations to better differentiate strains from different hosts. On comparison of the complete genomes of the bovine T320A and human Reiter strains of *T*. *phagedenis* there was a difference in size of 239.6kbp corresponding to 361 additional genes within the bovine strain. A BPGA pan-genome analysis compared the complete bovine and human *T*. *phagedenis* strains with *T*. *pallidum*. All three treponemes shared a core genome of 498 orthologs, with 274 genes identified as unique to bovine *T*. *phagedenis*, 116 to the human saprophyte and 495 unique to *T*. *pallidum*, with neither *T*. *phagedenis* strain individually sharing genes with *T*. *pallidum* (Fig 3A). Unique genes of bovine *T*. *phagedenis* strain T320A encoded functions for membrane transport, carbohydrate metabolism and genes implicated in infectious diseases (Fig 3C). The unique genes of the human strain Reiter encoded functions relating to carbohydrate metabolism, energy metabolism and replication and repair. Of note, the BDD-associated *T*. *phagedenis* strains (S4 Table), included genes encoding methyl accepting chemotaxis proteins, VirD4; a type IV secretion system protein, PstS; a phosphate substrate-binding protein and PhoU, which is involved in osmotic stress survival and enzymes for production of d-ManNAc3NAcA again.

**Fig 4:**
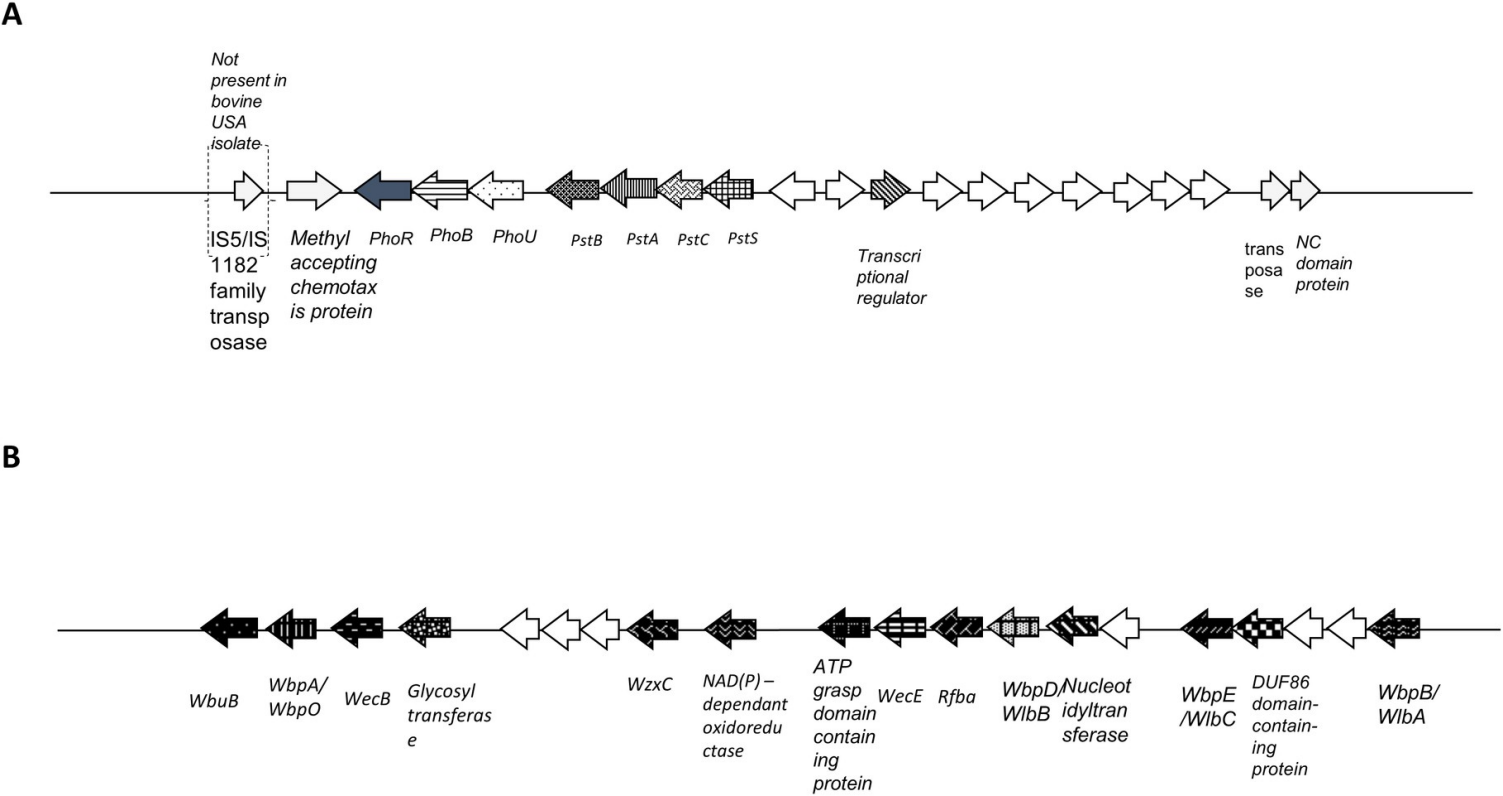
Genetic clusters unique to the bovine pathogenic *Treponema phagedenis* strains. The bovine *T*. *phagedenis* unique gene cluster contains two polycistronic operons for phosphate utilisation (**A**). All ORFs listed are present in UK, USA and Swedish *T*. *phagedenis* genomes and absent for human genomes except for area in dashed parentheses. The bovine *T*. *phagedenis* unique gene cluster containing a variety of genes associated with outer membrane production, nucleotide metabolism and oxidative stress (**B**). All ORFs present in UK, USA and Swedish *T*. *phagedenis* genomes and absent for human genomes. No mobile elements identified immediately surrounding these ORFs. ORFs not to scale. Arrows with solid white fill are hypothetical proteins.

Alignment of three bovine strains (from UK, Sweden and USA) and two human strains (USA and Germany) using a genome aligner (MAUVE) revealed many bovine strain specific genes of related functions were situated together in gene clusters (Figs 4 and S2 and S4 Table). The three bovine pathogenic strains from different geographic origins all shared these unique gene clusters encoding novel functions which were absent from the two human strains. These include a ~22.1 kb region containing two phosphate associated operons, with one containing a phosphate transporter system pstS, pstC, pstA, pstB and the other containing genes to control phosphate transport, i.e. phoU, phoB and phoR (Fig 4A and S4 Table) together with a proceeding methyl accepting chemotaxis protein. This unique gene cluster includes a further seven hypothetical proteins adjacent to phosphate utilisation regions ending with a transposase and NC domain protein with some bovine strains having a transposon element at the start also suggesting this region may be the result of horizontal gene transfer.

A ~53.3kb region encodes citric acid utilisation machinery and the aforementioned TraG/TraD/VirD4 as well as AbrB/MazE/SpoVT, several methyl transferases and Fic proteins, all typically considered as components of Type IV secretion systems, together with a fibronectin binding protein (S2 Fig and S4 Table). In the USA bovine strain, the fibronectin protein and ATP binding protein are replaced with a duf4868 protein. A hypothetical protein encompasses the region at the 5’ whilst 3’ finishes with a recombinase that it shares with the human strains.

A further ~19.5kb gene cluster contains genes associated with outer membrane production, nucleotide metabolism and oxidative stress (Fig 4B and S4 Table). This region does not have evidence of mobile genetic elements immediately surrounding it. Four proteins of the five-step biosynthesis pathway for d-ManNAc3NAcA (WbpA/WbpO, WbpB/WlbA, WbpE/WlbC, WbpD/WlbB) were found together in this bovine *T*. *phagedenis* gene cluster (Fig 4B and S4 Table) with the remaining WbpI/WlbD step present on another unique bovine strain gene cluster. This bovine strain region also contains a NADP oxidoreductase explaining the different number of oxidative stress genes in bovine and human *T*. *phagedenis* strains (Table 2).

To verify that observed genetic differences are consistently present in bovine strains and absent from human strains we surveyed a further nine bovine and two human strains for the presence of key genes within each genetic cluster (S5 Table) and identified a statistically significant association for the presence of these genes in bovine strains (S4 Table).

To further dissect pathogenesis, we compared the identified unique *T*. *phagedenis* locus tags from both data sets of 1) bovine DD versus GI treponemes and 2) human saprophyte *T*. *phagedenis* versus bovine DD strain. Only genes encoding WbpA/WbpO, WbpB/WlbA, WbpE/WlbC, WbpD/WlbB, and WbpI/WlbD appear associated with pathogenesis in both analyses.

### A genetic sequence duplication in bovine *T*. *medium* confers an expanded repertoire of stress related proteins compared with human *T*. *medium*

When comparing bovine T19 and human ATCC 700293^T^ *T*. *medium* genomes there was a difference of 164.6kbp corresponding to 155 additional genes within the bovine strain. There is a large genetic cluster present in bovine *T*. *medium* T19 and absent from human *T*. *medium* ATCC 700293^T^ measuring 162.3kb accounting for nearly the entire genome size difference. This region results from a genetic sequence duplication within bovine *T*. *medium*, resulting in an expanded repertoire of stress related proteins and a duplication of the Tetanolysin O toxin gene and bacterial Toxin:Antitoxin systems (Table 2). When comparing these *T*. *medium* strains with *T*. *pallidum* using a pan-genome analysis, 389 orthologs were shared (core) across all three treponemes with 32 unique genes in bovine *T*. *medium* T19, 57 in the human pathogen ATCC 700293^T^ and 602 unique to *T*. *pallidum*. Both the bovine and human *T*. *medium* each share a single unique ORF with *T*. *pallidum* due to corresponding pseudogenes (Fig 3B). Despite the larger genome size and greater number of genes, there are more unique genes in the human strain, probably resulting from the genome expansion being the result of a duplication. Unique bovine T19 strain genes encode functions including carbohydrate metabolism, membrane transport and cellular community (Fig 3C), whilst the human strain ATCC 700293^T^ encodes metabolism of carbohydrate, nucleotide and amino acids, replication and repair, signal transduction, cellular community and motility. Of note, the BDD treponeme pathogen unique genes include lsrA, an autoinducer- 2 (AI-2) transporter involved in quorum sensing and an additional NADP oxidoreductase, one of the oxidative stress genes differentiating bovine and human strains in Table 2. Human *T*. *medium* includes virulence associated genes such as methyl-accepting chemotaxis protein and the trkH, trkG, ktrB; trk system potassium uptake proteins, notably associated with *T*. *pallidum* pathogenesis. Most unique human *T*. *medium* genes appear as a result of pseudogenes in the bovine strain. Contrastingly, most unique genes in bovine strain T19 have no comparable human strain pseudogene.

### Identification of unique genes of bovine *Treponema pedis* and human *Treponema denticola* and comparison with *Treponema pallidum*

**Fig 5:**
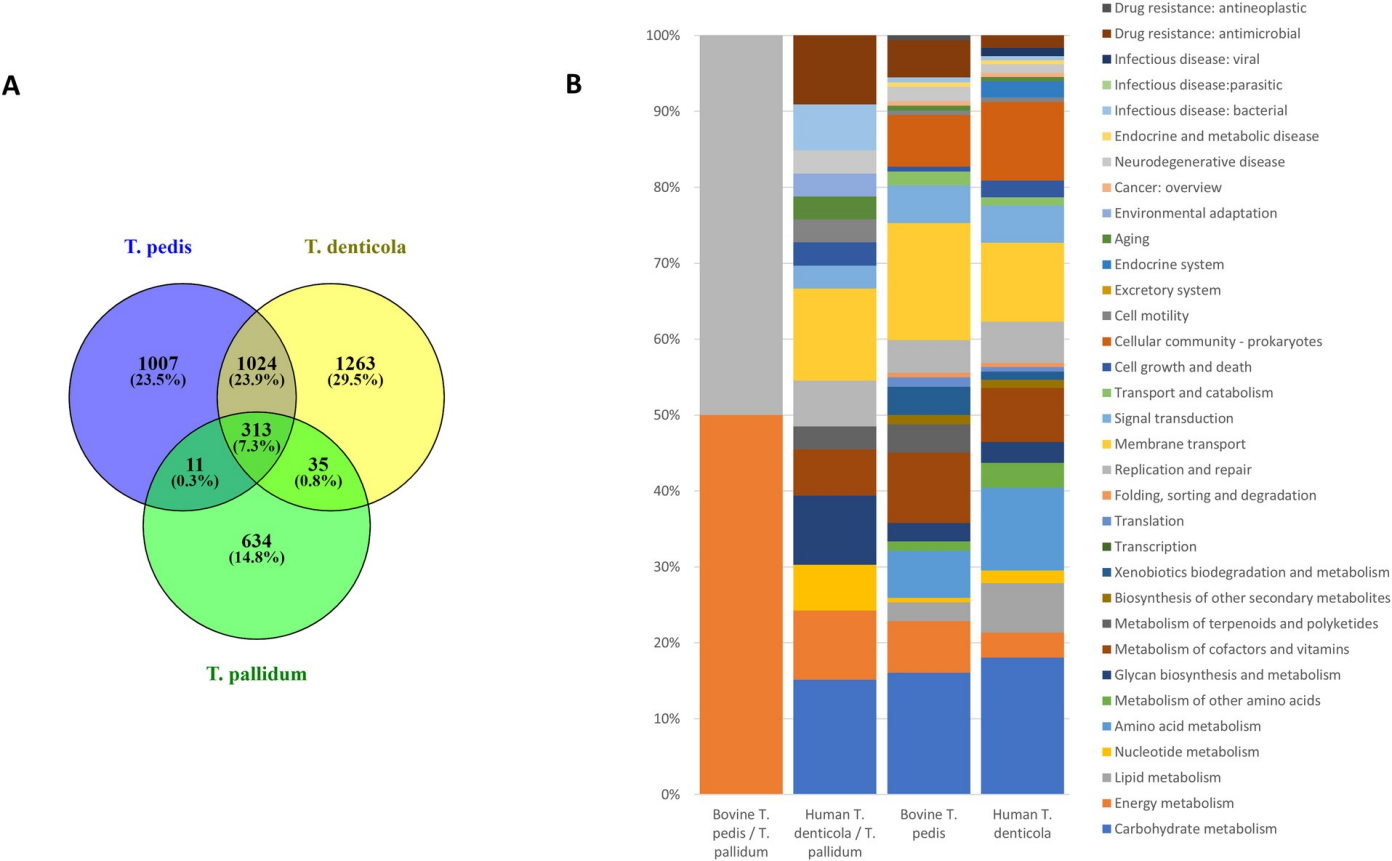
Core, accessory and unique genes shared between bovine pathogenic *Treponema pedis* and human pathogens *Treponema denticola* and *Treponema pallidum*. Venn diagram representation of the core, accessory and unique genes shared between bovine pathogenic *T*. *pedis* and human pathogens *T*. *denticola* and *T*. *pallidum* **(A)**. Numbers of shared gene families and/or unique genes are listed with the percentage of the total pan-genome this represents (for each comparison) listed in parentheses. Functional classification of genes unique to bovine pathogenic *T*. *pedis* and human pathogens *T*. *denticola* and *T*. *pallidum* or uniquely shared between either *T*. *pallidum* and either *T*. *pedis* or *T*. *denticola* (**B**). Only genes that could be assigned a KEGG category are represented in the bar graphs. Only 269 entries (26.7%) of the 1007 unique genes from **bovine *T*. *pedis*** could be annotated, only 318 entries (25.2%) of 1263 unique ***T*. *denticola*** genes could be annotated.: Only 8 entries (72.7%) annotated of the 11 uniquely shared genes between ***T*. *pallidum* / bovine *T*. *pedis*** could be annotated, only 28 entries (80.0%) annotated of 35 uniquely shared genes between ***T*. *denticola* / *T*. *pallidum*** could be annotated.

In comparisons of the complete genomes of bovine *Treponema pedis* T3552B^T^ with human *T*. *denticola* ATCC 35405 there was 46.2kb size difference, although the larger gene number belongs to *T*. *denticola*, which has 144 more. This contrast appears to arise because of the large number of pseudogenes in *T*. *pedis*, resulting from RHS genes and HTs (Table 2). A genetic cluster in *T*. *pedis* of 50.5kb consisting of a number of RHS and hypothetical proteins accounts in part for this difference. Pan-genome analyses to compare bovine *T*. *pedis* T3552B^T^ with human *T*. *denticola* and *T*. *pallidum*, exhibit a core genome of 313 orthologs across all three treponemes resulting in 1007 genes unique to the bovine *T*. *pedis*, 1263 unique to *T*. *denticola* and 634 to *T*. *pallidum*. Bovine *T*. *pedis* T3552B^T^ individually shared 11 unique genes with *T*. *pallidum* whilst human *T*. *denticola* individually shared 35 unique genes with *T*. *pallidum* (Fig 5A). Bovine *T*. *pedis* unique genes include those associated with energy metabolism, translation, folding, sorting and degradation, membrane transport, antimicrobial drug resistance, and metabolism of terponoids and polyketoids (Fig 5B). Human *T*. *denticola* differed by having unique genes encoding carbohydrate, lipid and amino acid metabolism and cellular community (Fig 5B). Uniquely shared functions only between *T*. *pallidum* and bovine *T*. *pedis* included energy metabolism and replication and repair whilst *T*. *denticola* uniquely shared genes with *T*. *pallidum* encoding carbohydrate and nucleotide metabolism, membrane transport, bacterial infectious diseases and drug resistance.

### Identification and production of putative outer membrane proteins shared across bovine digital dermatitis (pathogenic) cultivable treponemes

Whilst we identified a number of putative virulence mechanisms using comparative genomics, no identified protein candidates were predicted to be surface exposed but instead reside as inner membrane proteins or within the cytoplasm or periplasm (S3 Table). To better identify potential outer membrane proteins (OMPs) and protein vaccine candidates, we used a bioinformatic pipeline beginning with discovery of putative beta barrels. To identify putative OMPs the *T*. *medium* strain T19 was subjected to a previously described pipeline resulting in 182 potential OMPs identified by SignalP 4.1. Next, PRED-TMBB, BOMP and TMBETA-NET were used to filter the subset of sequences, and only those predicted to consist of a β-barrel tertiary structure by a minimum of one β-barrel prediction program and identified as present in all three bovine cultivable pathogenic treponemes, *T*. *medium*, *T*. *phagedenis* and *T*. *pedis* using a Markov cluster algorithm and BLAST were selected. This resulted in 15 putative OMP ortholog families across the three phylogroups (45 total) that were subjected to cloning (S6 Table) and subsequent expression trials. We successfully produced and purified one or more representatives from 10 of the ortholog families yielding 18 recombinant putative OMPs which were expressed with N-terminal polyhistidine-tag (Table 4). All of the produced OMPs except one (C5O78_01225) demonstrated Far-UV circular dichroism (CD) spectra consistent with a secondary structure predominantly comprising a β-sheet (S7 Table). Seven of the bovine *T*. *medium* orthologs shared greater than 95% amino acid sequence identity with the human *T*. *medium* ortholog, with the remaining three sharing greater than 89% sequence identity (Table 4). For bovine *T*. *phagedenis*, all ten sequences shared >99% amino acid sequence identity with the human non-pathogenic *T*. *phagedenis* strain. For *T*. *pedis*, all ten shared greater than 96.8% sequence identity with porcine *T*. *pedis*, although human orthologs for *T*. *denticola* ranged between 36.04–82.61%. When the putative OMP ortholog families were compared with the OMPs from the bovine GI tract to determine which were restricted to bovine DD treponemes, only ortholog families 8, 9 and 11 were bovine pathogen specific (Table 4).

**Table 4:** ECM binding of bovine digital dermatitis treponeme ortholog family proteins.

Ortholog Family	Locus tag	BDD *Treponema* phylogroup	Locus tags encoding the orthologous proteins from related human and porcine strains (% amino acid seq. identity)^1^	Host ligand^3^						Total ligands bound
				Laminin	Fibronectin	Collagen	Elastin	Chondroitin	Heparan sulphate	
1	C5N99_04710	*T*. *medium*	HMPREF9195_00920, 95.12% (ATCC 700293T)	-	+	+	+	+	-	4
	C5O78_02150	*T*. *phagedenis*	DWQ65_06125, 100.00% (Reiter)	n.d.	n.d.	n.d.	n.d.	n.d.	n.d.	-
	DYQ05_09320	*T*. *pedis*	TPE_1931,100% (A4) / TDE_1231,53.7% (ATCC 35405)	-	+	-	-	-	-	1
2	C5N99_04715	*T*. *medium*	HMPREF9195_00921, 99.45% (ATCC 700293T)	-	+	+	+	+	-	4
	C5O78_02155	*T*. *phagedenis*	DWQ65_06130, 100% (Reiter)	+	+	+	+	-	-	4
	DYQ05_09315	*T*. *pedis*	TPE_1930, 99.5% (A4) / TDE_1234, 46.8% (ATCC 35405)	n.d.	n.d.	n.d.	n.d.	n.d.	n.d.	-
3	C5N99_04785	*T*. *medium*	HMPREF9195_00935, 97.10% (ATCC 700293T)	-	-	-	+	+	+	3
	C5O78_07955	*T*. *phagedenis*	DWQ65_10640, 99.56% (Reiter)	n.d.	n.d.	n.d.	n.d.	n.d.	n.d.	-
	DYQ05_07395	*T*. *pedis*	TPE_1550, 99.5% (A4) / TDE_0014, 67.0% (ATCC 35405)	-	+	-	+	+	-	3
5	C5N99_05295	*T*. *medium*	HMPREF9195_01034, 94.81% (ATCC 700293T)	n.d.	n.d.	n.d.	n.d.	n.d.	n.d.	-
	C5O78_04000	*T*. *phagedenis*	DWQ65_12010, 99.80% (Reiter)	n.d.	n.d.	n.d.	n.d.	n.d.	n.d.	-
	DYQ05_01950	*T*. *pedis*	TPE_0412, 99.0% (A4) / TDE_2285, 49.2% (ATCC 35405)	+	+	-	+	+	+	5
6	C5N99_06860	*T*. *medium*	HMPREF9195_01342, 98.04% (ATCC 700293T)	n.d.	n.d.	n.d.	n.d.	n.d.	n.d.	-
	C5O78_01225	*T*. *phagedenis*	DWQ65_12610, 100% (Reiter)	+	+	-	+	-	-	3
	DYQ05_01600	*T*. *pedis*	TPE_0337, 98.5% (A4) / TDE_2598, 82.6% (ATCC 35405)	+	-	-	+	-	+	3
8	C5N99_06910	*T*. *medium*	HMPREF9195_01351, 89.36% (ATCC 700293T)	-	-	-	+	-	-	1
	C5O78_10020	*T*. *phagedenis*	DWQ65_10790, 99.49% (Reiter)	-	+	-	+	+	+	4
	DYQ05_06810^2^	*T*. *pedis*	TPE_1439, 99.0% (A4) / TDE_2308, 50.0% (ATCC 35405)	-	+	-	+	+	+	4
9	C5N99_03545	*T*. *medium*	HMPREF9195_00692, 97.02% (ATCC 700293T)	n.d.	n.d.	n.d.	n.d.	n.d.	n.d.	-
	C5O78_01255	*T*. *phagedenis*	DWQ65_12640, 100% (Reiter)	n.d.	n.d.	n.d.	n.d.	n.d.	n.d.	-
	DYQ05_09195	*T*. *pedis*	TPE_1910, 96.9% (A4) / TDE_1848, 58.9% (ATCC 35405)	+	+	-	-	+	-	3
11	C5N99_10335^2^	*T*. *medium*	HMPREF9195_01888, 99.59% (ATCC 700293T)	-	+	-	+	-	+	3
	C5O78_05585	*T*. *phagedenis*	DWQ65_04430, 99.59% (Reiter)	n.d.	n.d.	n.d.	n.d.	n.d.	n.d.	-
	DYQ05_13425^2^	*T*. *pedis*	TPE_2783, 98.5% (A4) / TDE_2674, 49.6% (ATCC 35405)	-	+	-	+	+	+	4
12	C5N99_10205	*T*. *medium*	HMPREF9195_01862, 95.33% (ATCC 700293T)	-	+	-	-	-	-	1
	C5O78_05635	*T*. *phagedenis*	DWQ65_04480, 100.00% (Reiter)	n.d.	n.d.	n.d.	n.d.	n.d.	n.d.	-
	DYQ05_12540	*T*. *pedis*	TPE_2593, 98.8% (A4) / TDE_1984, 55.9% (ATCC 35405)	-	-	-	-	-	-	0
15	C5N99_02965^2^	*T*. *medium*	HMPREF9195_00579, 94.25% (ATCC 700293^T^)	-	-	-	+	-	+	2
	C5O78_04920	*T*. *phagedenis*	DWQ65_08260, 100.00% (Reiter)	n.d.	n.d.	n.d.	n.d.	n.d.	n.d.	-
	DYQ05_07390	*T*. *pedis*	TPE_1549, 99.4% (A4) / TDE_1666, 36.1% (ATCC 35405)	n.d.	n.d.	n.d.	n.d.	n.d.	n.d.	-

^1^ Strain designation listed in parentheses. ATCC700293: human *T*. *medium*, Reiter: human *T*. *phagedenis*, A4: Porcine *T*. *pedis* and ATCC35405: *T*. *denticola*.

^2^Adhesion data previously reported [71].

^3^n.d. Not determined as we were unable to effectively express the encoded protein.

### Animal and human cultivable pathogenic treponemes have a wide range of genes encoding ECM adhesins

Spirochete OMPs have frequently been implicated in attachment to host extra-cellular matrix (ECM) molecules [38,39]. Here, the ability of the 18 recombinant, refolded proteins, representing 10 ortholog families, to bind to a panel of six ECM ligands was investigated by an ELISA-based system (Table 4). A negative control host protein, BSA, was selected, against which binding of recombinant treponeme proteins was quantified by ELISA using the ECM ligands as the target on the microplates. Interactions were considered specific when a statistically significant (P<0.05) difference was reached (Table 4). Amongst recombinant proteins tested, elastin, followed by fibronectin (n = 14 and n = 13, respectively) were the most frequently identified binding ligands. Collagen was bound by only three of the recombinant proteins tested and was thus the most infrequent binding partner. Only one recombinant protein (DYQ05_12540) did not adhere to any ECM components surveyed and all but three (DYQ05_09320, C5N99_06910 and C5N99_10205) were multispecific (mode = 4 ligands; maximum = 5 ligands). Complete concordance in ligand specificity was not demonstrated between any of the orthologous proteins, although these analyses revealed that a considerable overlap in specificity exists. For example, the family 2 orthologues, C5N99_04715 and C5O78_02155, both exhibited adherence to the following core ECM components: fibronectin, collagen and elastin. However, C5N99_04715 additionally adhered to chondroitin and C5O78_02155 additionally adhered to laminin. Similar observations were made for ortholog OMP families 3, 8 and 11 (Table 4). A failure to express or adequately purify a number of these proteins precluded several ortholog comparisons.

### A specific adhesin ortholog family enables discrimination of diseased animals

Using an ELISA-based serological assay, we sought to evaluate the systemic IgG antibody response to the putative treponemal OMPs in dairy cows with a recent (≤6 month) history of BDD diagnosis. Relative to healthy control animals, these data (Table 5) reveal that the majority of recombinant proteins tested (n = 16; 88.9%) were not recognised by either specific IgG1 or IgG2 antibodies in sera from cows with BDD. However, the mean IgG2 OD values obtained in the BDD-exposed group were significantly higher than the mean OD values obtained in the BDD non-exposed group for two putative OMPs, namely, the *T*. *medium* phylogroup C5N99_06910 (P ≤ 0.005) and its *T*. *pedis* ortholog, DYQ05_06810 (P ≤ 0.005). Whilst the *T*. *phagedenis* ortholog (C5O78_10020) did have a greater IgG2 serotitre in infected animals, this was not statistically significant. By determining a negative cut-off value, these data demonstrated that 91.6% and 75% of infected animals elicited specific IgG2 antibodies to C5N99_06910 and DYQ05_06810, respectively. Thus, out of the ten ortholog families analysed, only one family (ortholog family 8, Table 5) was shown capable of eliciting IgG2 antibodies following natural infection, whilst IgG1 seroreactivity was not detected against any of the recombinant proteins tested. In fact, a statistically significant (P ≤ 0.05) reduction in the mean ELISA OD of BDD-positive cows, relative to healthy controls, was observed against five recombinant proteins, suggesting that in these instances, a reduction in antibody titre was measured. This phenomenon was restricted to IgG1 serotitres and was observed for one putative OMPs from the *T*. *phagedenis* (C5O78_01225) and four putative OMPs from the *T*. *medium* (C5N99_04715, C5N99_10335, C5N99_10205 and C5N99_02965).

**Table 5:** Serological assessment of IgG1 and IgG2 responses to putative treponemal OMP ortholog family members by ELISA.

Ortholog Family	Locus tag	BDD phylogroup	Expression identified^1^	IgG1 serology^2^			IgG2 serology^2^		
				Naïve	Exposed	*P* value	Naïve	Exposed	*P* value
1	C5N99_04710	*T*. *medium*	Y	0.353 ±0.118	0.302 ±0.080	0.5335	0.275 ±0.134	0.188 ±0.080	0.3137
	C5O78_02150	*T*. *phagedenis*	Y	n.d.	n.d.	n.d.	n.d.	n.d.	n.d.
	DYQ05_09320	*T*. *pedis*	Y	0.174 ±0.062	0.176 ±0.045	0.7401	0.147 ±0.045	0.172 ±0.062	0.4828
2	C5N99_04715	*T*. *medium*	Y	1.109 ±0.168	0.905 ±0.265	0.0429*	0.852 ±0.363	1.064 ±0.484	0.3054
	C5O78_02155	*T*. *phagedenis*	N	0.248 ±0.097	0.258 ±0.107	0.5787	0.186 ±0.111	0.206 ±0.120	0.9823
	DYQ05_09315	*T*. *pedis*	Y	n.d.	n.d.		n.d.	n.d.	
3	C5N99_04785	*T*. *medium*	Y	0.121 ±0.050	0.165 ±0.045	0.0862	0.099 ±0.041	0.114 ±0.032	0.3847
	C5O78_07955	*T*. *phagedenis*	Y	n.d.	n.d.	n.d.	n.d.	n.d.	n.d.
	DYQ05_07395	*T*. *pedis*	Y	0.352 ±0.109	0.354 ±0.076	0.7733	0.220 ±0.085	0.239 ±0.102	0.7726
5	C5N99_05295	*T*. *medium*	Y	n.d.	n.d.	n.d.	n.d.	n.d.	n.d.
	C5O78_04000	*T*. *phagedenis*	Y	n.d.	n.d.	n.d.	n.d.	n.d.	n.d.
	DYQ05_01950	*T*. *pedis*	Y	0.342 ±0.059	0.358 ±0.150	0.6526	0.197 ±0.057	0.306 ±0.142	0.2376
6	C5N99_06860	*T*. *medium*	N	n.d.	n.d.		n.d.	n.d.	
	C5O78_01225	*T*. *phagedenis*	Y	0.213 ±0.026	0.163 ±0.049	0.0149*	0.256 ±0.072	0.285 ±0.087	0.7726
	DYQ05_01600	*T*. *pedis*	N	0.282 ±0.036	0.232 ±0.835	0.0590	0.205 ±0.084	0.296 ±0.191	0.1551
8	C5N99_06910	*T*. *medium*	Y	0.126 ±0.016	0.133 ±0.037	0.9770	0.082 ±0.010	0.184 ±0.054	0.0006**
	C5O78_10020	*T*. *phagedenis*	Y	0.363 ±0.131	0.309 ±0.095	0.3427	0.189±0.070	0.219 ±0.073	0.2199
	DYQ05_06810^3^	*T*. *pedis*	Y	0.084 ±0.017	0.090 ±0.027	0.4828	0.067 ±0.006	0.111 ±0.031	0.0020**
9	C5N99_03545	*T*. *medium*	Y	n.d.	n.d.		n.d.	n.d.	
	C5O78_01255	*T*. *phagedenis*	Y#	n.d.	n.d.		n.d.	n.d.	
	DYQ05_09195	*T*. *pedis*	Y	0.394 ±0.085	0.274 ±0.089	0.0999	0.270 ±0.080	0.273 ±0.077	0.5833
11	C5N99_10335^3^	*T*. *medium*	Y	0.448 ±0.105	0.320 ±0.117	0.0299*	0.335 ±0.136	0.242 ±0.124	0.1509
	C5O78_05585	*T*. *phagedenis*	Y	n.d.	n.d.		n.d.	n.d.	
	DYQ05_13425^3^	*T*. *pedis*	Y	0.144 ±0.069	0.180 ±0.075	0.4716	0.099 ±0.044	0.155 ±0.072	0.1337
12	C5N99_10205	*T*. *medium*	Y#	1.528 ±0.292	1.144 ±0.473	0.0226*	0.923 ±0.333	1.230 ±0.758	0.6650
	C5O78_05635	*T*. *phagedenis*	Y	n.d.	n.d.		n.d.	n.d.	
	DYQ05_12540	*T*. *pedis*	Y	0.911 ±0.157	0.883 ±0.350	0.7726	0.556 ±0.134	0.874 ±0.449	0.1485
15	C5N99_02965^3^	*T*. *medium*	Y	0.191 ±0.022	0.133 ±0.010	0.027*	0.074 ±0.007	0.090 ±0.007	0.220
	C5O78_04920	*T*. *phagedenis*	Y	n.d.	n.d.		n.d.	n.d.	
	DYQ05_07390	*T*. *pedis*	N	n.d.	n.d.		n.d.	n.d.	

^1^Identification of expression by the respective treponemal species (when grown in routine culture) as determined by Nano LC MS/MS analysis. All putative OMPs detected in a minimum of two peptide identifications except # which only had one.

^2^Error values indicate standard errors of the means. Asterisks indicate a significant difference in IgG seroreactivity relative to control sera as determined by Mann-Whitney U test (*, P < 0.05; **, P < 0.005). n.d. Not determined as we were unable to effectively express the encoded protein.

^3^Seroreactivity data previously reported [71].

### Proteomic analyses

To confirm genetic similarities and differences observed by comparative genomics were actually reflected in protein expression, we completed a proteomic analysis of the six treponemal strains for which we had generated complete genomes under normal culture conditions. This proteomic analysis revealed that of the unique gene clusters within bovine *T*. *phagedenis*, we observed expression of several members of the phosphate utilisation gene cluster including PhoU (S4 Table). Furthermore, we failed to detect expression of the putative Type IV secretion system, although the five-enzyme biosynthetic pathway for the production of d-ManNAc3NAcA was expressed in bovine *T*. *phagedenis* (S4 Table).

In terms of the novel virulence factors identified that were shared between the bovine pathogens and absent from bovine commensal treponemes (Tables 3 and S3), the majority of their identified genes were expressed across the pathogenic DD treponemes including the five-enzyme biosynthetic pathway for the production of d-ManNAc3NAcA which was expressed by all three bovine DD treponemes with the exception of one of the enzymes in the pathway not being detected in *T*. *pedis*.

In terms of the 18 OMPs that we produced as recombinants, 14 were identified as actually expressed by respective treponemal strains including ortholog family 8 which have promise as diagnostic molecules and families 9 and 11 which together with family 8 are the only bovine DD treponeme specific OMP ortholog families (Table 5).

### Osmotic stress response discriminates bovine and human *Treponema phagedenis*

**Fig 6:**
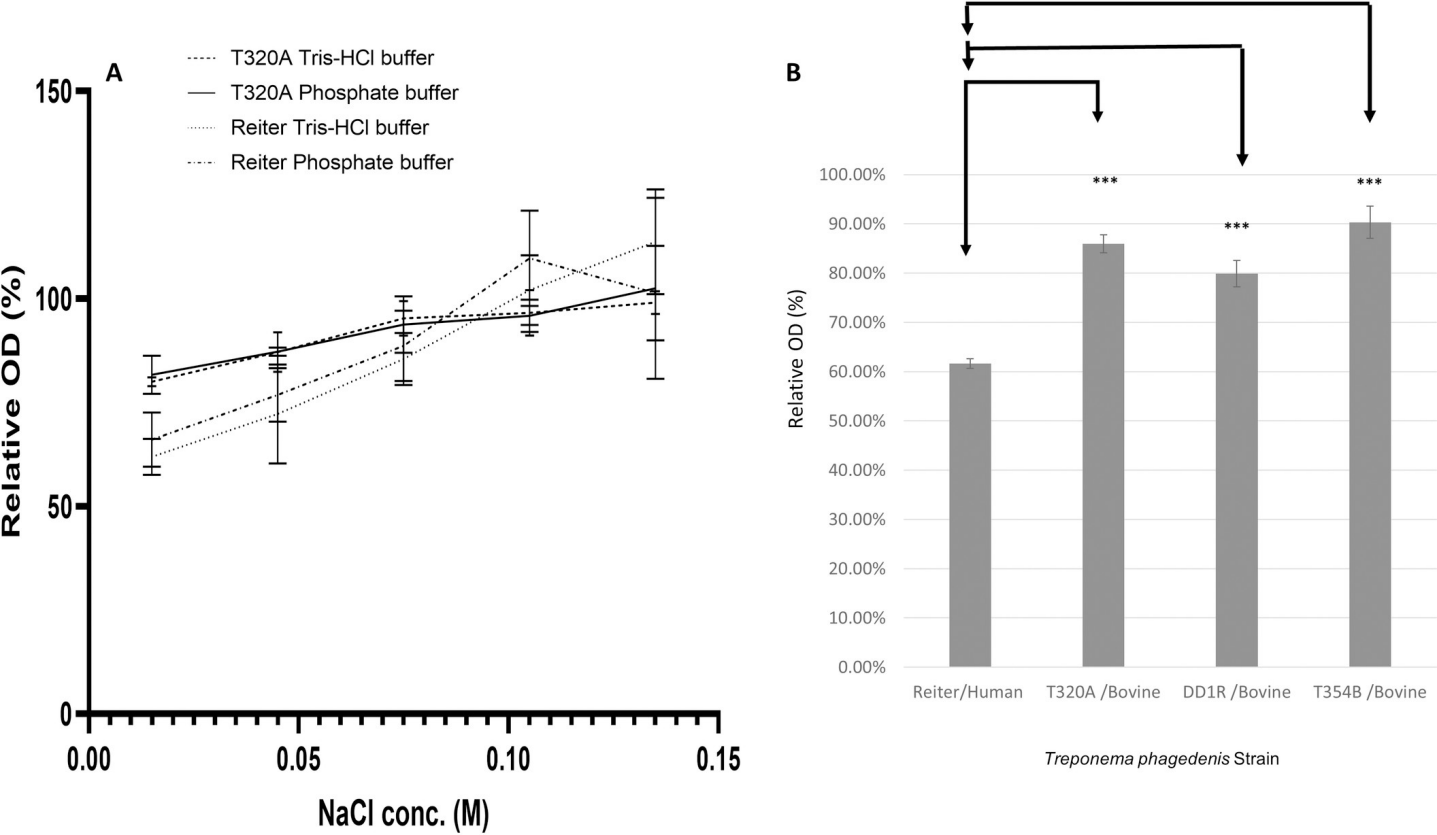
Survival of human and bovine strains of *Treponema phagedenis* when exposed to osmotic stress. Survival as determined by relative turbidity of human and bovine strains of *T*. *phagedenis* when suspended in solutions of NaCl below physiological concentrations (0.015 M, 0.045 M, 0.075 M, 0.105 M and 0.135 M) after 90 minutes incubation at 37 degrees C (**A**). Bars represent standard error of the mean. Data are representative of three experimental repeats undertaken on different days (each consisting of two technical replicates). Relative optical density of four *T*. *phagedenis* strains post-exposure to distilled deionised water for 90 minutes incubation at 37 degrees C (**B**). Bars represent standard error of the mean. Data are representative of four experimental repeats undertaken on different days (each consisting of three technical replicates).

Given that the bovine *T*. *phagedenis* strains had clear genetic differences in content relating to cell structure and survival, especially given the genetic presence and expression of PhoU, we investigated whether osmotic stress could differentiate bovine and human strains of *T*. *phagedenis*. We compared a human and bovine strain across a range of salt concentrations using bacterial turbidity as a measure of survival [40] which demonstrated a decrease in turbidity, indicative of an increase in degenerative cells, when osmotic strength was reduced below typical host physiologic concentration. There was no significant difference for each strain although bovine *T*. *phagedenis* had a higher relative turbidity at 0.015M NaCl, the lowest molarity analysed, compared with the human *T*. *phagedenis* (Fig 6A and S8 Table). To consider this difference in survivability at low osmotic strength further, we investigated treponemal turbidity change in deionised, distilled water. For multiple *T*. *phagedenis* strains it was found the human Reiter treponeme had the lowest relative OD of 61.65% compared to PBS control (Fig 6B and S8 Table) whilst each of the three bovine DD strains had at least a 20% increased survival in water in comparison. The relative OD differs significantly between the four *T*. *phagedenis* strains analysed (one-way ANOVA: P <0.0001). A post hoc Tukey multiple comparison test was used to discriminate any statistical significance for individual differences. The relative OD of the human *T*. *phagedenis* Reiter with each of the bovine strains differed significantly (S8 Table, P < 0.001 for each bovine strain).

## Discussion

Here we describe the complete genomes of several cultivable pathogenic and commensal treponemes, as well as their associated proteomes, which should be a substantial resource for future use by the scientific community. Sequencing of these genomes together with key phenotyping, has allowed for substantial comparisons to be made, to better understand the evolution of pathogenesis within this important spirochetal genus.

In terms of genome content, there appears to be a substantial GC content difference between the syphilis treponemes and the most closely related cultivable treponemes. This GC content difference coincides with a five or ten-fold reduction in A and T HTs and a moderate increase in G and C HTs in the syphilis treponemes. Indeed *T*. *pallidum* is circa 1Mb in size, three fold smaller than its closest relatives from the cultivable treponemes, *T*. *medium* and *T*. *phagedenis* (circa 3Mb), and yet has the largest number of G and C tracts. The presence of G and C HTs and their ability to undergo slipped strand mispairing in the syphilis treponemes has been reported [41,42]. That so many A and T HTs were lost suggests that the genome decay that produced *T*. *pallidum* may have been expedited by, or targeted to, these A and T HTs tracts. In contrast with the pathogenic bovine treponemes and human pathogens having large numbers of HT tracts, the commensal *T*. *ruminis* had the least number of HTs. Interestingly, another pathogenic spirochete, *Borrelia burgdorferi*, has a significant overrepresentation of poly A and T HTs [43]. Indeed, expansion/contraction of HTs has been reported to play a key function for pathogenic bacteria during adaptation to unique environments [44].

Interestingly, whilst the core-genome phylogenetic trees (Fig 1A and S1 Fig) led to the two syphilis treponemes forming their own cluster, the pan genome tree clustered the bovine DD treponemes with the syphilis treponemes (Fig 1B). This is in agreement with historic reports that the human derived closest relatives of bovine DD treponemes, especially *T*. *phagedenis*, have substantial cross reactivity to antibodies generated against to the agent of syphilis [19,45]. The coding density was lower for the bovine pathogenic treponemes (80.7–84.6%) than for human *T*. *denticola*, the two syphilis treponemes and the *T*. *ruminis* genomes (90.7–93.2%). Both human *T*. *medium* and *T*. *phagedenis* also had marginally higher coding density than the BDD treponemes. These differences coincide with differences in the number of pseudogenes with the bovine pathogens having a greater number in each relevant comparison. Comparisons of BDD and GI tract treponemes also identified a greater number of uniquely shared genes involved with replication and repair amongst the bovine pathogenic treponemes. Together with evidence for mobile genetic elements enabling key determinants associated with survival and pathogenesis (Fig 4 and S2 Fig), it appears mobile genetic elements are likely bringing about genome decay in the bovine pathogens with gene inactivation occurring through multiple recombination events as described for the bovine spirochetal pathogen *Leptospira borgpetersenii* [46]. For that pathogenic bovine leptospire it was considered mobile genetic element derived events increased host (bovine) dependence as a result of a reduction in the range of genes involved in nutrient acquisition. A feature here appears to be the bovine pathogens had a greater number of membrane transport genes whilst simultaneously exhibiting a reduction in diversity of functions also (Figs 2, 3 and 5). Mobile genetic elements integrating novel determinants and causing genome decay is frequently a driver of host dependency and niche adaptation [47]. Given there was a greater number of oxidative stress associated genes in each of the bovine pathogenic treponemes compared to human pathogens and commensals (Tables 2 and S1 and Fig 4) this would suggest that acquisition of oxidative stress genes through mobile genetic elements had enabled an ecological niche switch from restriction to anaerobic niches (i.e. periodontal pockets and GI tracts) to colonisation and invasion of the bovine skin and transmissibility within a dairy farm environment.

Initial comparisons investigated recognized *T*. *pallidum* and *T*. *denticola* virulence factor distribution across the bovine pathogenic treponemes. Only two hemolysins (TlyC and HlyIII) and the Filament protein (CfpA) discriminated both the syphilis treponemes and bovine and human cultivable pathogens from *T*. *ruminis* with TlyC previously described as a marker of treponeme pathogenesis [35,48]. Based on orthology criteria applied using Mauve and BPGA with a 50% threshold, a key *T*. *pallidum* virulence determinant, pallilysin (Tp0751) [49] was restricted to the two syphilis treponemes. Orthologs from other treponemes have been described for pallilysin although they exhibit low sequence identity and restricted function [49]. The pallilysin polycistronic partner Tp0750 was present in all treponemes except *T*. *ruminis* and therefore appears to be a further marker of treponeme virulence.

To identify putative virulence mechanisms we used pan-genomic analyses of bovine pathogens and commensals to identify pathogen specific genes. Functional annotation of pathogen associated genes included those encoding lipid and nucleotide metabolism, translation, replication and repair, membrane transport, cell community and motility (Fig 2E). Complete pathways for pyruvate oxidation to acetyl-CoA and fatty acid metabolism through beta-oxidation to produce acyl-CoA were identified as unique across bovine pathogens. We previously reported BDD treponemes as serum dependent [35] and considered whether they should be assigned as asaccharolytic akin to human *T*. *denticola* and *T*. *vincentii* [50]. Such treponemes preferentially ferment amino acids and can only degrade glucose using the Embden-Meyerhof pathway as a minor energy source [51]. In contrast, many GI commensal treponemes are reported to be saccharolytic and are named after their sugar-fermenting activities [35]. Interestingly, it would appear here, that this core shared metabolic pathway for glucose metabolism, including pyruvate kinase (Table 3), appears implicated in pathogenesis. Interestingly, pyruvate kinase is implicated in papillomavirus pathogenesis [52] and it is of note that BDD is alternatively termed papillomatous DD based on pathological presentation [53,54]. In line with amino acid fermentation, other key ortholog families unique to the BDD pathogens included four peptidases. The chaperone GroEL which is important in pathogenesis of legionellosis and tuberculosis [55,56], was identified as unique to bovine pathogenic treponemes and previously has been implicated as a BDD treponeme virulence factor [57]. Additional putative virulence markers in the bovine pathogens include peptidyl-prolyl cis-trans isomerase A (PPIA; cyclophilin A) which has been linked to virulence in *Staphylococcus aureus* [58]. There was an absence of signal peptidase 2 (for processing surface lipoproteins) within the bovine GI treponemes, with only the bovine pathogens containing this protein, with cell surface lipoproteins considered key to the pathogenesis of pathogenic spirochetes [59]. Given absence of signal peptidase 2 has not previously been reported in prokaryotes, future studies are needed to confirm this finding. Finally, key enzymes involved in O-Antigen nucleotide sugar biosynthesis (WbpA/WbpO, WbpB/WlbA, WbpE/WlbC, WbpD/WlbB, WbpI/WlbD) were also uniquely present in the pathogens and not the commensal representatives. These genes encode a five-enzyme biosynthetic pathway for the production of d-ManNAc3NAcA, a rare di-*N*-acetylated mannuronic acid sugar residue found in the LPS of *Bordetella pertussis*, *Bordetella bronchiseptica* and certain serogroups of *Pseudomonas aeruginosa* [60]. In these pathogens, their LPS is an important virulence factor enabling survival within the host through immune evasion, including protection from serum-mediated killing [61,62]. Interestingly, β-D-mannuronic acid is a recognised anti-inflammatory [63]. Furthermore, we have recently demonstrated that *T*. *ruminis*, a bovine GI treponeme identified here as not having the pathway for d-ManNAc3NAcA, triggers a much greater inflammatory host response than the three BDD treponeme taxa in an *in vitro* fibroblast tissue culture model, where it significantly dysregulated over three times the number of host mRNA transcripts compared to BDD treponemes [64]. Intriguingly, extracted *T*. *medium* glycoconjugate has previously been identified as inhibiting human gingival fibroblasts activation when stimulated with periodontopathic bacteria extracts [65]. This immunomodulating characteristic was attributed to the lipophilic portion of the glycoconjugate and was also identified as reducing LPS-induced, but not TNF-induced, human monocyte activation [66].

When we compared the genomes of *Treponema phagedenis* strains representing bovine pathogenic strains and the non-pathogenic human saprophyte there was a substantial size difference (circa 240kB) attributed to several large genetic clusters. These genetic clusters were conserved across both UK bovine strains (S4 Table) and bovine strains from different countries worldwide (UK, Sweden and USA) and absent from human strains from the USA, Germany and Russia (S4 Table and Figs 4 and S2). On investigation of these genetic clusters, one cluster (Fig 4B and S4 Table) contained the same key difference as that identified between the bovine pathogens and GI commensals, namely the enzyme pathway for or d-ManNAc3NAcA. It is intriguing that these two different comparisons implicate the same biosynthetic pathway in the pathogenesis of the genus *Treponema*.

A second bovine pathogen unique genetic cluster from *T*. *phagedenis* (S2 Fig and S4 Table) included TraG/TraD/VirD4, AbrB/MazE/SpoVT, methyl transferases and Fic (filamentation induced by cyclic AMP) proteins, which are frequently components of Type IV secretion systems, as well as a predicted fibronectin binding protein. Interestingly, horizontal acquisition of a VirD4 type IV secretion system (T4SS) has a key role in *Bartonella* species host adaptation and is responsible during infection for exporting a plethora of effector proteins which subvert host pathway including diverse cellular and innate immune functions which enables systemic pathogen spread [67]. Furthermore, genomic islands encoding T4SS and fic domain proteins are considered responsible for the pathogenicity of *Campylobacter fetus* [68].

A third unique bovine *T*. *phagedenis* gene cluster contained phosphate transport genes (pstS, pstC, pstA, pstB) and genes for phosphate transport control phoU, phoB and phoR (Fig 4A and S4 Table). Given phoU mutants in *P*. *aeruginosa* resulted in poor growth and increased stress sensitivity including to osmotic stress [69], we investigated whether the bovine and human strains differed in response to osmotic stress. When we investigated survival ability using turbidity [40], we were able to show that a human and bovine *T*. *phagedenis* strain differed in survival at low salt concentrations. Subsequently, when several bovine *T*. *phagedenis* strains and the human strain were compared for survival in water alone, bovine strains differing significantly (S5 Table, P < 0.001 for each) with at least a 20% increased survival compared to the human *T*. *phagedenis*. This suggests that as well as *phoU* being expressed (S4 Table), it affords bovine *T*. *phagedenis* a survival advantage, thus facilitating transmission of this BDD treponeme and may explain why it can contribute to a surface skin lesion rather than being restricted to the GI or genitourinary tract. There has been much discussion with regards to whether *T*. *medium* and *T*. *phagedenis* strains derived from separate host species (bovines or humans) represent diverged species, including the use of the ‘–like’ suffix for the bovine strains from these treponemal species [6,31]. Whilst we have been able to designate a *T*. *denticola*-like species as *T*. *pedis* [30], *T*. *phagedenis* and *T*. *medium* from the different host species have been designated as indistinguishable and within the same phylogroup or species [37,70]. The data presented here for the first time indicates that the *T*. *phagedenis* strains derived from different host may in fact be different subspecies, which should underpin future additional polyphasic phenotyping and associated taxonomic appraisals.

To further dissect treponeme pathogenesis and identify vaccine candidates for the severe cattle disease, BDD, we determined which genes were predicted to encode outer membrane beta-barrel proteins across the three cultivable BDD treponeme phylogroups/species. We successfully cloned, expressed and refolded representatives from ten ortholog families across the three phylogroups and subjected them to host-ligand binding surveys, identifying that the majority bind to ECM molecules. Indeed, the majority of these putative OMPs appear to be multi-specific adhesins with diversity of functions exhibited both between and within ortholog families. Given the high sequence identity of several of these proteins to orthologs in nearest human relative strains, it should be considered we have also characterised several putative OMPs from human treponeme pathogens also. Interestingly, whilst host adhesion is considered key to virulence, the human non-pathogenic *T*. *phagedenis* strain had many of these adhesins with no or limited sequence diversity, suggesting this saprophyte could also bind host ECM, blurring the lines between saprophyte and pathogen. However, we have also demonstrated a greater number of survival orientated genes, an enhanced survival phenotype, as well as a putative secretion system enabling differentiation of the bovine pathogenic *T*. *phagedenis* from the human non-pathogenic saprophyte.

When subjected to ELISA against sera from infected BDD and healthy cattle, we identified one putative OMP ortholog family as a serodiagnostic antigen target for BDD infection with the *T*. *medium* ortholog enabling a 91.6% specificity, greater than the 75% for the *T*. *pedis* ortholog (the latter being recently reported by us [71]). Despite substantial evidence for *T*. *phagedenis* involvement in BDD, serologically its ortholog was only towards significance, and therefore the poorer diagnostic candidate of the three. Supporting this, when comparing the identified OMP ortholog families against predicted OMPs from bovine GI treponemes only three OMP ortholog families (Table 5 families 8, 9 and 11) were absent from the commensal treponemes with the putative OMP ortholog family of serodiagnostic value being one of these. A further unique observation was that for IgG1 antibodies several OMPs exhibited a statistically significant (P ≤ 0.05) reduction in antibody titre in BDD-positive cows, relative to healthy controls. This observation is fascinating given we have identified a putative BDD treponeme biosynthetic pathway for a novel sugar with immunomodulatory properties [60]. Indeed, more generally, human cultivable treponemes can evade both innate and adaptive host immune responses [66] and if these potentially hidden OMPs can be unveiled to the host immune response they may offer an alternative route to vaccination against this severe infectious disease of cattle.

Here we have described a range of putative pathogenicity mechanisms, which likely enable bovine (and human) pathogenic treponemes to cause disease. We describe a range of survival apparatus mechanisms that appear to be specific to the bovine pathogens and likely enable them to live on the skin surface, unlike human periodontal pathogens or saprophytes. It is intriguing that having a substantial extended repertoire of survival genes might enable bovine *T*. *phagedenis* to become a pathogen, although the presence of a putative secretion system needs further study. One BDD treponeme OMP ortholog family appears to have serodiagnostic promise and would also be worthy of investigation as a trivalent vaccine for BDD. The bovine pathogenic treponemes appear to share a five-enzyme biosynthetic pathway for the production of a rare di-*N*-acetylated mannuronic acid sugar with potential immunomodulatory activity, and this may explain why some OMPs counterintuitively have reduced serotitres in infected animals. This work has shed light on treponeme host adaptation and has identified candidate molecules for future diagnostics, vaccination and therapeutic intervention.

## Methods

### Ethics statement

Sampling was in accordance with UK legislation and approved by both a UK Home Office Project License PPL 70/8330 and the University of Liverpool Ethical Review Process with application number VREC111.

### Treponeme isolates

Treponeme strains, *T*. *medium*-like strain T19, *T*. *phagedenis*-like T320A and *T*. *pedis* T3552B^T^ were isolated from UK cattle BDD lesions and grown as described previously [31]. Human oral pathogen *T*. *medium* strain ATCC 700293^T^ was isolated from human subgingival dental plaque in Japan [10] and the considered saprophytic, non-pathogenic human derived *Treponema phagedenis* biotype Reiter was isolated from the genito-urinary tract in Germany [19], with each grown within the same media as their respective bovine species representatives. The bovine commensal *Treponema ruminis* Ru1^T^ was isolated from a cattle rumen [35] and cultured as previously described [14]. DNA was extracted from these cultures using a Wizard HMW extraction Kit (Promega, Southhampton, UK), following manufacturer’s instructions. The obtained DNA concentrations were examined using a Qubit 2.0 Fluorometer and NanoDrop ND-2000 spectrophotometer (both from Thermo Fisher Scientific, Loughborough, UK) to assess purity.

### Genome generation and annotation

Genome DNA library construction and sequencing was performed as described for *Arsenophonus nasoniae* [72]. Briefly, DNA preparation was achieved by producing a standard fragment and paired end single stranded DNA template using the GD DNA library Preparation Kits (Roche Applied Sciences, USA). These fragments (400-600bp standard fragment and 2.5Kb paired end) were amplified by emulsion PCR and sequenced on a GS-FLX (454 Life Sciences, Roche Applied Sciences, USA). The resulting genomes were in multiple contigs, with some large gaps, and due to the lack of a suitable reference genome sequence the same DNA extractions were used for further sequencing using the Illumina Mi-Seq platform (Illumina, USA). Here, standard paired end and mate pair sequencing libraries were prepared according to manufacturer’s protocols and sequenced on one flowcell of the MiSeq 2000 at 2x250 bp paired-end sequencing with v2 chemistry. Initial processing and quality assessment of the sequence data was performed using in-house pipelines at the University of Liverpool Centre for Genomic Research (University of Liverpool, Liverpool, UK). Reads from paired-end and mate-pair libraries for each isolate were assembled together to make single genome assemblies using SPAdes version 3.0.0 [73], using the k-mer values for 2x250bp read pairs: 21, 33, 55, 77, 99, 127. Subsequently, the two sets of sequencing reads for each genome were assembled with Newbler (v1.1.03.24) to produce hybrid assemblies of Roche GS-FLX sequencing reads and Illumina paired-end reads providing genome coverage and characteristics as described in S9 Table. To obtain complete genome sequences, remaining gaps were closed using Sanger PCR walking between sequences. The assembled genomes was annotated using the NCBI Prokaryotic Genome Annotation Pipeline [74] and manually curated. Genomes sequenced as part of this study, together with relevant treponeme genomes for comparison, are listed in Table 1, including respective Genbank accession numbers.

### Pan- and core-analyses of genomes and inference of function

The bacterial pan-genome pipeline BPGA (version 1.3) was used to carry out pan- and core-analyses [75]. Core genomes were obtained from whole genomes by applying the USEARCH program (version 9.0) using a 50% sequence identity criteria [76]. For each comparison to visualise core, accessory and unique genes we inputted ortholog family lists outputted by BPGA into Venny V2.0 (3http://bioinfogp.cnb.csic.es/tools/venny/). To investigate mechanisms of pathogenesis, we compared the three BDD pathogen species (Table 1) with three bovine GI tract species including *T*. *ruminis* (Table 1) and using additional draft bovine treponemes *T*. *rectale* [16] and *T*. *bryantii* [15] with Genbank accession numbers CP031517 and PRJEB17384 respectively. To further dissect functions associated with pathogenesis or commensalism we compared the three BDD pathogen species genomes with complete genomes from bovine *T*. *ruminis* and human *T*. *pallidum* (Table 1).

In each analysis either the unique or relevant accessory genome ortholog families were submitted to BlastKOALA [77] for functional annotation according to KEGG orthology (KO). Subsequently, KEGG annotated ortholog families were reconstructed to metabolic and regulatory pathways using KEGG Mapper [78] producing BRITE hierarchies and KEGG modules based on K number assignment enabling high-level function comparisons and identification of novel pathways. High-level functional assignment data were imported into Excel (Microsoft, Redmond, Washington, USA) and converted to bar charts for graphical representation.

Identification of eukaryotic-like domains and secretion systems used EffectiveELD [79]. For comparative genomics a progressiveMauve alignment was implemented using Mauve [80] with 50% ortholog sequence identity and the ortholog list exported for interrogation. Identification of oxidative stress associated genes used lists of oxidative stress genes from comprehensive oxidative stress surveys including that of the Serratia sp. LCN16 genome, bioleaching acidophiles and *T*. *pallidum* [81–83].

### Identification of shared pathogenic mechanisms of the bovine digital dermatitis treponemes

To identify whether known treponeme virulence determinants could differentiate bovine pathogens from commensals/saprophytes, a progressiveMauve alignment was produced using the ten genomes from various hosts listed in Table 1 and an ortholog list outputted. Locus tags of known *T*. *pallidum* or *T*. *denticola* virulence factors were used to determine ortholog presence using the genome alignment and outputted ortholog list. *Treponema denticola* virulence factors included previous descriptions [84] and comprised 13 genes. *Treponema pallidum* virulence factors included 31 genes as previously defined [85] as well as Pallilysin (Tp0751) and associated protein Tp0750 [49].

To identify novel disease associated determinants to differentiate bovine pathogenic and commensal treponemes, the pan genome analysis of three bovine pathogen species and three bovine commensals was carried out. Orthologs uniquely shared by the BDD pathogens or bovine commensals were identified from the accessory genome using Venny V2.0 and subjected to BlastKOALA for functional annotation according to KEGG orthology (KO). Resulting lists of annotated genes for the pathogens and commensals were outputted into Excel and manually curated. Novel ortholog families were further verified using the Mauve alignment and ortholog list.

### Identification of unique gene clusters differentiating treponemal strains from different host species

Genome comparisons to contrast bovine and human *T*. *phagdenis* and then bovine and human *T*. *medium* used the completed genomes, subjected to BPGA as above. Subsequently, progressive Mauve alignments were produced and interrogated using the unique genes identified by BPGA, to investigate genomic differences in terms of location and underlying mechanisms (evidence of transposases etc). Given the number of unique genes localising as clusters within the bovine *T*. *phagedenis* strain we included additional draft genomes in the Mauve alignment including bovine strains from Sweden (V1: GCA_000944995.1) and the USA (4A: GCA_000513775.1), together with an additional human strain from the USA (F0421: GCA_000187105.1) allowing for comparison of strains from diverse geographical locations. To confirm presence/absence of unique genetic clusters we developed PCR primers for select genes within each cluster (S5 Table) using Primer3 [86] and investigated the presence of these genes within a further nine UK bovine strains including *T*. *phagedenis* strains T354B, T116B, G169A, W35, T136, DD1R, T380, T323C F1 and T2721A and two human strains Kazan 8 and CIP62.29 [70]. Polymerase chain reaction used Taq polymerase (Qiagen, Manchester, UK) according to manufacturer’s instructions with thermal cycles of 95°C for 5 min; 35 cycles of 95°C for 1 min, 59°C for 3 min and 72°C for 3 min; and 72°C for 5 min. Strains T320A and Reiter were used as positive and negative controls respectively with presence of amplification products determined by agarose gel electrophoresis according to standard protocols.

### Genome phylogenetic analyses

A pan-genome phylogenetic tree of available treponeme genomes (from Genbank) was reconstructed with the unweighted pair group method with arithmetic mean (UPGMA) algorithm using a binary presence/absence gene pan matrix produced from BPGA [75] using orthologous clusters generated from USEARCH. A core-genome phylogenetic tree of available treponeme genomes was constructed using protein sequences from 20 random orthologous gene clusters, sequences aligned by MUSCLE [87], concatenated and an UPGMA phylogenetic tree constructed. For a RiboMLST phylogenetic analysis [36], ribosomal genes were obtained from all available treponeme genomes and a maximum likelihood tree generated using Mega 7.0 [88], and the general time reversible model, as determined by Topali [89] with bootstrapping using 100,000 iterations.

### *In silico* identification of OMPs

Genomes of *T*. *medium* T19, *T*. *phagedenis* T320A and *T*. *pedis* T3552B^T^ (Table 1) were analysed *in silico* to identify putative OMPs via prediction of encoded β -barrel structural motifs as described previously [71]. Briefly, putative coding sequences (CDS) of the *T*. *medium* T19 genome were translated into their amino acid sequences using Artemis [90] and submitted to SignalP 4.1 [91] to identify all sequences harbouring a signal peptidase I cleavage site. All CDS predicted to contain a signal peptidase I cleavage site were further scrutinised for amino acid signatures suggestive of a β-barrel structure using BOMP [92], TMBETA-NET [93], and PRED-TMBB [94]. All CDS features predicted to contain a β-barrel motif by at least one prediction program were retained. Orthologs of putative *T*. *medium* T19 OMPs were identified in *T*. *phagedenis* T320A and *T*. *pedis* T3552B^T^ genomes using a combination of a Markov cluster algorithm [95] and BLAST [96] and β-barrel designations verified as above.

### Cloning and expression of candidate antigens

Gene cloning used the Gateway System (Life Technologies, Paisley, UK). Putative OMP sequences (with removed signal peptide) were PCR-amplified from genomic DNA using high-fidelity Phusion polymerase (Thermo Scientific, Hemel Hempstead, UK). Amplification primers (S6 Table) included a CACC overhang to facilitate directional cloning into entry plasmid, pENTR/d-TOPO (Life Technologies, Paisley, UK). One Shot TOP10 Chemically Competent *E*. *coli* cells were transformed with the constructs and cultured in accordance with manufacturer’s instructions and as described previously [71]. Plasmid DNA was isolated with Qiagen Plasmid Miniprep Kit (Qiagen, Manchester, UK) and inserts transferred to Gateway pDEST17, via site-directed integration and submitted for Sanger sequencing to verify gene inserts.

### Protein expression, refolding, and purification

Protein expression, refolding and purification was performed as described previously [71]. Briefly, protein expression was performed in BL21(DE3) Competent Cells (Life Technologies, Paisley, UK), transformed with relevant pDEST17-gene constructs and induced by addition of 1mM isopropyl-D-thiogalactopyranoside (IPTG; Sigma-Aldrich, Gillingham, UK). Inclusion bodies (IBs) were harvested by centrifugation, washed twice and resuspended in solubilization buffer (6 M guanidine hydrochloride, 50mM Tris-HCl [pH 7.9], and 1mM EDTA; 40ml per 500mg of IB) with insoluble material removed by centrifugation. Soluble fraction was added dropwise to a refolding buffer (250mM NaCl, 50mM Tris-HCl [pH 7.9], 5% N,N-dimethyldodecylamine N-oxide solution (LDAO; Sigma-Aldrich, Dorset, UK) and dialysed against 10 volumes of dialysis buffer (250mM NaCl, 50mM Tris-HCl [pH 7.9], 0.1% LDAO). Metal affinity chromatography was used to purify the refolded recombinant proteins, as described previously. Recombinant protein eluents were filter sterilised (0.2um), purity assessed by SDS-PAGE and stored at –80°C. Far-UV circular-dichroism (CD) spectroscopy was performed as previously described [71] and spectra interpreted using BestSel [97].

### Evaluation of immunogenicity during natural infection

The IgG1 and IgG2 seroreactivity to purified recombinant proteins was assessed for sera from 16 adult Holstein-Friesian cows with a recent (6-month) history of BDD, collected from a dairy herd in Cheshire, UK. Sera of cows (n = 5) with no history of BDD, living in a closed dairy herd in Monmouthshire, UK, were included as a control group. Sera preparation and ELISA were performed as described previously [71]. Briefly, sera were added to ELISA plates coated with individual recombinant proteins. Bound bovine IgG1 and IgG2 antibodies were detected by the primary antibodies, mouse anti-bovine IgG1 and mouse anti-bovine IgG2 antibody (Sigma-Aldrich, Dorset, UK), respectively, followed by the secondary antibody, goat anti-mouse horseradish peroxidase (HRP)-conjugated IgG (Sigma-Aldrich, Dorset, UK). TMB substrate was added to all wells, followed by 0.5M HCl to terminate colorimetric reaction. Optical densities (OD) were read at 450nm using a microplate reader (Multiskan EX; Thermo, Loughborough, UK). All data were analysed in GraphPad Prism 5 (GraphPad, San Diego, CA) using the Mann Whitney U test.

### Evaluation of adhesin function

Here, ELISAs were used to screen recombinant proteins (diluted 10 μg/ml in PBST) for ability to attach to individual ECM components using a previously described method [71]. All ECM macromolecules were from Sigma-Aldrich (Dorset, UK): collagen I from bovine skin, elastin from bovine neck filament, heparan sulfate from bovine kidney, chondroitin sulfate from bovine cartilage, and laminin-1 from basement membrane mouse sarcoma. Recombinant proteins were added to microplate wells coated with individual ECM components. Bound recombinant proteins were detected using primary antibody, mouse anti-polyhistidine IgG antibodies (Sigma-Aldrich, Dorset, UK) and secondary antibody, horseradish peroxidase (HRP)-conjugated goat anti-mouse IgG (Sigma-Aldrich, Dorset, UK). TMB substrate was added, then 0.5M HCl to terminate colorimetric reaction and OD read at 450nm. Statistical analysis compared the ELISA ODs for the negative control, BSA, with those for components of the ligand panel, using One-way ANOVA and Dunnett’s Multiple Comparison Test.

### Proteomic analyses

Treponeme cultures were grown as previously described [35] and then centrifuged at 10,000 ***g*** for 5 minutes, supernatant removed and pellet washed twice in PBS. Bacterial pellets were lysed in 4% (w/v) SDS detergent, 100mM DTT in 100mM Tris-HCl buffer [pH 7.6]. and heated at 95°C for 10 minutes. This followed by 3 cycles of sonication on ice (Vibra-cell 130PB sonicator, 20Hz, with microprobe, 20 second alternating sonication/rest intervals). Samples were centrifuged at 16,000 ***g*** for 10 minutes. The supernatant was retained and protein concentration determined using a detergent compatible Bradford protein assay (ThermoFisher, UK). Digestion used the FASP method [98] with samples normalized to 100 μg total protein in 200μl 8M urea, 100mM Tris-HCl buffer [pH 7.6]. Proteins were alkylated with 15mM iodoacetamide (Sigma) and SDS removed by washes with 8M urea, 100mM Tris-HCl [pH 7.6], and 50mM ammonium bicarbonate. Proteins were digested with proteomic-grade trypsin (50:1 protein:trypsin) and incubated at 37°C overnight. Eluted peptides were acidified by adding TFA to a final concentration of 0.5% (v/v). Peptide samples were desalted and fully evaporated with a centrifugal evaporator (Eppendorf). Each sample was reconstituted in 0.1% (v/v) TFA, 3% (v/v) methanol and stored at -80°C until analysis.

Nano LC MS/MS analysis was performed as described [99]. Peptides were analysed by on-line nanoflow LC using the Ultimate 3000 nano system (Dionex/Thermo Fisher Scientific). Samples were loaded onto a trap column (Acclaim PepMap 100, 2 cm × 75 μm inner diameter, C18, 3 μm, 100 Å) before separation by the analytical column (Easy-Spray PepMap RSLC 50 cm × 75 μm inner diameter, C18, 2 μm, 100 Å) fused to a silica nano-electrospray emitter (Dionex). Column operation was at 30°C and the LC system was coupled to a Q-Exactive mass spectrometer (Thermo Fisher Scientific). Chromatography used a buffer system consisting of 0.1% formic acid (buffer A) and 80% acetonitrile in 0.1% formic acid (buffer B). Peptides were separated by linear gradient of 3.8–50% buffer B over 90 minutes at a flow rate of 300 nl/min. The Q-Exactive was operated in data-dependent mode with survey scans acquired at a resolution of 70,000 at m/z 200. Top 10 most abundant isotope patterns with charge states +2 to +5 from survey scan were selected with an isolation window of 2.0Th and fragmented by higher energy collisional dissociation with normalized collision energies of 30. Maximum ion injection times for the survey scan and the MS/MS scans were 250 and 50 ms, respectively, and ion target value set to 1E6 for survey scans and 1E5 for MS/MS scans. MS/MS events acquired at a resolution of 17,500. Repetitive sequencing of peptides was minimized through dynamic exclusion for 20s.

Spectral MS data were analysed using PEAKS studio 10 software (Bioinformatics Solutions Inc., Waterloo, ON, Canada). Tandem MS data were searched against predicted protein sequences for each treponeme. Search parameters included precursor mass tolerance of 15ppm and fragment mass tolerance at 0.02 Da. Two missed tryptic cleavages were permitted. Carbamidomethylation (cysteine) was set as a fixed modification and oxidation (methionine) set as a variable modification. Protein score (-10lgP) of greater than 20 required for identification. False discovery rate was at 1%. Results were filtered to include only proteins present in two or more replicates and with greater than two unique peptides per protein.

### Osmotic stress assay

Here, 750μL aliquots of *T*. *phagedenis* treponemes, T320A and Reiter (at 1.14 × 10^8^ cells/ml) at late exponential phase were centrifuged at 5000*g* for 5 minutes. Cells were washed in 1.4ml Phosphate Buffered Saline (PBS; Sigma-Aldrich, [pH 7.4] 0.137 M NaCl), re-centrifuged and re-suspended in test solutions. Test solutions included a range of NaCl concentrations (0.015 M, 0.045 M, 0.075 M, 0.105 M and 0.135 M) in two different buffers; 0.1M Tris-HCl [pH 7.4] and 0.1M Potassium Phosphate Buffer [pH 7.4]. Bacterial suspensions in test solutions were incubated for 90 minutes at 37°C, which has previously been reported to produce a measurable osmotic stress response for *T*. *phagedenis* Reiter [40]. Control test buffer consisted the bacteria resuspended in PBS (Sigma-Aldrich, [pH 7.4] 0.137 M NaCl). Resuspended treponemes were analysed to quantify conversion to degenerative state by measuring the accompanying reduction in OD as described [40]. Here, OD measurements used a UV/Visible spectrophotometer (Ultrospec 2000, Pharmacia Biotech, Uppsala, Sweden) at 540 nm wavelength and converted to relative OD values compared with control. Three experimental repeats were undertaken on different days for each test solution. Subsequently, a water survival assay carried out for four strains of *T*. *phagedenis*; including the human Reiter and bovine T320A, DD1R and T354B strains [70], where after bacterial preparation washing, a test solution of distilled, deionised H_2_O was incubated and relative OD measured as above. Mean, standard error of mean and statistical significance were calculated and analysis of variance (ANOVA, α = 0.05) was completed on the Reiter and T320A data to determine any difference due to strain or buffers used. A one-way ANOVA test (α = 0.05) was used to identify significant difference between survival of the four different strains of *T*. *phagedenis* when resuspended in water with post hoc Tukey’s multiple comparisons test applied to determine any significant difference in survivability between strains.

## Supporting information

S1 Table Oxidative stress related genes in the genome sequenced treponemes and important relatives. (DOC)

S2 Table Distribution of shared *T*. *denticola* and *T*. *pallidum* virulence associated genes across the disease associated and GI treponemes. (DOC)

S3 Table Identified disease associated determinants differentiating bovine pathogenic and commensal treponemes. (DOC)

S4 Table Unique genes with putative functions attributed to bovine and human *Treponema phagedenis* and their presence within relevant human and bovine strains. (DOC)

S5 Table Primers used to confirm the distribution of select genes within gene clusters across *Treponema phagedenis* strains. (DOC)

S6 Table Primers used for putative OMP gene amplification to enable gene cloning and expression. (DOC)

S7 Table Secondary structure and fold recognition based on circular dichroism spectrometry of treponeme putative OMPs. (DOC)

S8 Table Relative optical density of four *T*. *phagedenis* strains post-exposure to distilled deionised water. (DOC)

S9 Table Genome assembly characteristics of the assembled genomes. (DOC)

S1 Fig A RiboMLST phylogenetic tree of relevant treponemes. Ribosomal gene sequences were obtained, aligned and concatenated and a maximum likelihood tree generated using Mega 7.0 (88), the general time reversible model, as determined by Topali [89] with bootstrapping using 100,000 iterations.
(TIF)

S2 Fig The bovine *Treponema phagedenis* unique gene cluster containing Type IV secretion system components, a citrate utilisation cluster, fibronectin binding protein and cell filamentation proteins. The bovine *T*. *phagedenis* unique gene cluster containing Type IV secretion system components, a citrate utilisation cluster cell filamentation proteins with either a fibronectin binding protein (A: UK and Sweden bovine strains) or a duf4868 protein (B: USA bovine strain). All ORFs present in UK, USA and Swedish *T*. *phagedenis* genomes and absent for human genomes except USA bovine strain has no ATP binding protein and fibronectin binding protein and instead has a duf4868 containing protein.
(TIF)

S1 File Presence of eukaryotic-like domains (ELD) across key human and animal treponemes. Presence of eukaryotic-like domains (ELD) across human and bovine representatives of *T*. *medium* and *T*. *phagedenis*, bovine *T*. *pedis*, bovine *T*. *ruminis*, human *T*. *denticola*, porcine *T*. *pedis* and human and rabbit syphilis treponemes. Comparative strains from the same bacterial species but different host species are listed together with differences in domain presence denoted by PFAM accession being highlighted in bold. For each ELD the respective genome locus tag, ELD PFAM domain and respective Z score are listed [79].
(XLSX)
