# Supplementary material for: Dissecting the molecular diversity and commonality of bovine and human treponemes identifies key survival and adhesion mechanisms
Source: PLoS Pathog. 2021 Mar 29;17(3):e1009464. doi: 10.1371/journal.ppat.1009464 (PMC8049484; doi:10.1371/journal.ppat.1009464)
Supplement: S1 Table — (DOC) [file ppat.1009464.s001.doc]

**S1 Table 1. Oxidative stress related genes in the genome sequenced treponemes and important relatives.**

|  | **Gene^1^** | ***Treponema medium* ATCC 700293^T^** | ***Treponema medium***  **DSM 18689** | ***Treponema phagedenis***  **Strain Reiter** | ***Treponema phagedenis***  **DSM 18690** | ***Treponema pedis***  **DSM 18691^T^** | ***Treponema pedis***  **strain T A4** | ***Treponema ruminis***  **DSM 103462^T^** | ***Treponema denticola***  **ATCC 35405** | ***T. pallidum*** ***subsp. pallidum (Nichols)*** | ***T. paraluiscuniculi strain***  ***Cuniculi A*** |
| --- | --- | --- | --- | --- | --- | --- | --- | --- | --- | --- | --- |
| Host |  | human | bovine | human | Bovine | Bovine | porcine | bovine | human | human | rabbit |
| ROS | KatG/KatE/KatN | -/-/- | -/-/- | -/-/- | -/-/- | -/-/- | -/-/- | -/-/- | -/-/- | -/-/- | -/-/- |
| Scavenging | SOD: Fe.Mn/Cu.Zn/Ni | -/-/- | -/-/- | -/-/- | -/-/- | -/+/- | -/+/- | -/-/- | -/-/- | -/-/- | -/-/- |
|  | Dfx | - | - | + | + | + | + | - | + | + | + |
|  | Rbr/Rub | -/+ | -/+ | -/+ | -/+ | +/+ | +/+ | 3+/- | +/+ | -/+ | -/+ |
|  | Fdx | 3+ | 3+ | + | + | 2+ | 2+ | 2+ | 2+ | - | - |
|  | PFOR/IorA | +/- | +/- | +/- | +/- | +/- | +/- | +/- | +/- | +/- | +/- |
|  | NifJ | + | + | 2+ | 2+ | + | + | + | + | - | - |
|  | PAP/ PAP2 | +/+ | 2+/+ | +/2+ | +/2+ | +/2+ | +/2+ | +/3+ | +/2+ | +/2+ | +/2+ |
|  | GSHPx/ Glutaredoxin | +/- | +/- | -/- | -/- | +/- | +/- | 2+/- | +/- | -/+ | -/+ |
|  | Fld | 4+ | 4+ | 3+ | 3+ | 4+ | 4+ | 8+ | 4+ | + | + |
|  | NADH Oxidase | - | - | - | - | 2+ | 2+ | - | + | + | + |
|  | FMN dehydrogenase | + | + | + | + | + | + | - | + | + | + |
|  | NADP Oxidoreductase | 4+ | 5+ | 5+ | 6+ | 4+ | 4+ | 3+ | 3+ | - | - |
|  | FAD Oxidoreductase | 3+ | 3+ | 2+ | 2+ | 5+ | 5+ | - | 4+ | - | - |
|  | FAD/NADP oxidoreductase | 2+ | 2+ | 2+ | 2+ | + | + | + | + | - | - |
|  | Gfo,Idh,MocA Oxidoreductase | + | + | 1+ | 2+ | + | + | 3+ | - | - | - |
|  | UcpA | - | - | - | - | + | + | - | - | - | - |
| Protein | TrxA/TrxB | +/2+ | +/2+ | +/+ | +/+ | +/+ | +/+ | +/- | +/+ | +/+ | +/+ |
| Repair | Tpx/ Trx dependent Tpx | -/- | -/- | +/+ | +/+ | -/- | -/- | -/- | -/- | -/- | -/- |
|  | Thioredoxin family protein | + | + | + | + | + | + | - | + | + | + |
|  | MsrA/MsrB | -/+ | -/+ | -/+ | -/- | -/+ | -/+ | +/- | -/+ | +/- | +/- |
|  | Hsp33 | + | + | + | + | + | + | + | + | - | - |
| Lipid Repair | AhpC or Peroxiredoxin | + | + | + | + | 2+ | 2+ | + | + | + | + |
|  | AhpF/Ohr/Dyp | -/-/- | -/-/- | -/-/- | -/-/- | -/-/- | -/-/- | -/-/- | -/-/- | -/-/- | -/-/- |
| DNA Repair | UvrABC complex | +/-/- | +/-/- | +/-/- | +/-/- | +/-/- | +/-/- | +/-/- | +/-/- | +/-/+ | +/-/+ |
|  | MutL/MutS/MutS2 | +/+/+ | +/2+/2+ | +/+/+ | +/+/+ | 2+/+/+ | 2+/+/+ | +/+/+ | 2+/+/+ | +/+/- | +/+/- |
|  | DNA glycosilases | 3+ | 3+ | 3+ | 3+ | 2+ | 2+ | 2+ | - | 2+ | 2+ |
|  | Photolyases/Dps | -/- | -/- | -/- | -/- | -/- | -/- | -/- | -/- | -/- | -/- |
|  | RecA/RadA | +/+ | +/+ | +/+ | +/+ | +/+ | +/+ | +/+ | +/+ | +/+ | +/+ |
| Regulators | LexA/Fur/Rex | -/-/+ | -/-/+ | -/-/+ | -/-/+ | -/-/+ | -/-/+ | -/-/+ | -/-/+ | -/-/- | -/-/- |
|  | Metal dependent regulator | + | + | 2+ | 2+ | 2+ | 2+ | + | + | + | + |
|  | PerR/ Irr /OxyR | -/-/- | -/-/- | -/-/- | -/-/- | -/-/- | -/-/- | -/-/- | -/-/- | -/-/- | -/-/- |
|  | OhrR/Spx/Sta1 | -/-/- | -/-/- | -/-/- | -/-/- | -/-/- | -/-/- | -/-/- | -/-/- | -/-/- | -/-/- |
| Total Counts |  | 42 | 46 | 43 | 44 | 49 | 49 | 42 | 40 | 24 | 24 |

^1^Identification of oxidative stress associated genes used lists of oxidative stress genes from comprehensive oxidative stress surveys including those of Serratia sp. LCN16 genome, bioleaching acidophiles and T. pallidum . **Abbreviations.** Catalases include KatG, KatE, KatN: catalases G, E and N; superoxide dismutases include various SOD: superoxide dismutase and Dfx: Desulfoferrodoxin (superoxide reductase); Rbr: rubrerythrin; Rub: rubredoxin; Fdx: ferredoxin; PFOR: pyruvate-ferredoxin oxidoreductase; IorA: indolepyruvate ferredoxin oxidoreductase subunitalpha; Fld: flavodoxin; NifJ: pyruvate-flavodoxin oxidoreductase; peroxidases include PAP1 and 2: phosphatidate phosphatase 1 and 2 and GSHPx: Glutathione peroxidase as well as those related to the thioredoxins; Thioredoxins include TrxA/TrxB: thioredoxin system; Tpx: thiol peroxidase; MsrA/B: methionine sulfoxide reductases; AhpC, AhpF: alkyl hidroperoxide reductase subunits; Ohr: organic hydroperoxide reductase; Dyp: dyp peroxidise; MutL/S: components of mismatch repair system; RecA/RadA: repair protein; LexA: SOS response regulator; Dps: DNA binding protein involved in protection; Fur family: fur family protein; PerR, Irr, OxyR, SoxRS, OhrR, Spx: Sta1: oxidative stress responsive proteins; Rex: Redox-responsive repressor.

**References:**

1. Cárdenas JP, Moya F, Covarrubias P, Shmaryahu A, Levicán G, Holmes DS, et al. Comparative genomics of the oxidative stress response in bioleaching microorganisms. Hydrometallurgy. 2012;127-128:162-7.

2. Radolf JD, Deka RK, Anand A, Šmajs D, Norgard MV, Yang XF. Treponema pallidum, the syphilis spirochete: making a living as a stealth pathogen. Nat Rev Microbiol. 2016;14(12):744-59.

3. Vicente CS, Nascimento FX, Ikuyo Y, Cock PJ, Mota M, Hasegawa K. The genome and genetics of a high oxidative stress tolerant Serratia sp. LCN16 isolated from the plant parasitic nematode Bursaphelenchus xylophilus. BMC Genomics. 2016;17:301.
