# Supplementary material for: Dissecting the molecular diversity and commonality of bovine and human treponemes identifies key survival and adhesion mechanisms
Source: PLoS Pathog. 2021 Mar 29;17(3):e1009464. doi: 10.1371/journal.ppat.1009464 (PMC8049484; doi:10.1371/journal.ppat.1009464)
Supplement: S2 Table — (DOC) [file ppat.1009464.s002.doc]

**S2 Table. Distribution of shared *T. denticola* and *T. pallidum* virulence associated genes across the disease associated and GI treponemes**

|  | ***Treponema medium* ATCC 700293^T^** | ***Treponema medium***  **DSM 18689** | ***Treponema phagedenis***  **Strain Reiter** | ***Treponema phagedenis***  **DSM 18690** | ***Treponema pedis***  **DSM 18691^T^** | ***Treponema pedis***  **strain T A4** | ***Treponema ruminis***  **DSM 103462^T^** | ***Treponema denticola***  **ATCC 35405** | ***Treponema pallidum*** ***subsp. pallidum (Nichols)*** | ***Treponema paraluiscuniculi strain***  ***Cuniculi A*** |
| --- | --- | --- | --- | --- | --- | --- | --- | --- | --- | --- |
| Host | human | bovine | Human | bovine | Bovine | porcine | bovine | human | human | rabbit |
| Chymotrypsin-like protease/Dentilisin (PrtP) (TDE0760) | + | + | - | - | + | + | - | + | - | - |
| Dentilisin associated proteins PrcA/PrcB (TDE0761/TDE0762) | +/- | +/- | -/+ | -/+ | +/+ | +/+ | -/- | +/+ | -/- | -/- |
| Protease II (PtrB) (TDE2140) | - | - | - | - | + | + | - | + | - | - |
| Prolyl oligopeptidase (PreP) (TDE1195) | - | - | - | - | + | + | - | + | - | - |
| Major Outer Sheaf Protein (Msp) (TDE0405) | - | - | - | - | - | - | - | + | - | - |
| Surface antigen (TDE2258) | - | - | - | - | + | + | - | + | - | - |
| Cystalysin/Hemolysin (Hly) (TDE1669) | + | + | + | + | + | + | - | + | - | - |
| Hemolysin C (TlyC/HlyC) (TDE1243) | + | + | + | + | + | + | - | + | + | + |
| Chemotaxis protein A (CheA) (TDE1491) | + | + | + | + | + | + | + | + | + | + |
| Flagellar hook protein (FlgE)(TDE2768) | + | + | + | + | + | + | + | + | + | + |
| Filament protein (CfpA) (TDE0842) | + | + | + | + | + | + | - | + | + | + |
| Ig-like protein (TDE0362) | - | - | - | - | - | - | - | + | - | - |
| Pallilysin (Tp0751)/ associated protein (Tp0750) | -/+ | -/+ | -/+ | -/+ | -/+ | -/+ | -/- | -/+ | +/+ | +/+ |
| Tpr A/B/C/D]  (TPAMA_0009/TPAMA_0011/TPAMA_0117/TPAMA_0131) | -/-/-/- | -/-/-/- | -/-/-/- | -/-/-/- | -/-/-/- | -/-/-/- | -/-/-/- | -/-/-/- | +/+/+/+ | +/+/+/+ |
| hypothetical outer membrane protein (tp92) (TPAMA_0136) | - | - | - | - | - | - | - | - | + | + |
| M23B subfamily peptidase (TPAMA_0155) | - | - | - | - | - | - | - | - | + | + |
| lipoprotein, 15 kDa (tpp15) (TPAMA_0171) | - | - | - | - | - | - | - | - | + | + |
| conserved hypothetical protein (fibronectin-like) (TPAMA_0263) | - | - | - | - | - | - | - | - | + | + |
| Tpr protein E/F/G  (TPAMA_0313/TPAMA_0316/TPAMA_0317) | -/-/- | -/-/- | -/-/- | -/-/- | -/-/- | -/-/- | -/-/- | -/-/- | +/+/+ | +/+/+ |
| Outer membrane protein (tp92) (TPAMA_0326) | - | - | - | - | - | - | - | - | + | + |
| Tpr protein H/I/J/K/L (TPAMA_0610/TPAMA_0620/TPAMA_0621/TPAMA_0897/TPAMA_1031) | -/-/-/- | -/-/-/- | -/-/-/- | -/-/-/- | -/-/-/- | -/-/-/- | -/-/-/- | -/-/-/- | +/+/+/+ | +/+/+/+ |
| O-sialo glycoprotein endopeptidase (TPAMA_0680) | - | - | - | - | - | - | - | - | + | + |
| laminin-binding protein (TPAMA_0751) | - | - | - | - | - | - | - | - | + | + |
| bacterioferrin (TPAMA_1038) | - | - | - | - | - | - | - | - | + | + |
| IIISP family Type III (virulence-related) secretory pathway proteins (FliF/FliH/FliI/ flhA/ flhB/fliR/fliQ/fliP) (TPAMA_0399/TPAMA_0401/TPAMA_0402/TPAMA_0714-TPAMA_0718) | +/+/+/+/+/+/+/+ | +/+/+/+/+/+/+/+ | +/+/+/+/+/+/+/+ | +/+/+/+/+/+/+/+ | +/+/+/+/+/+/+/+ | +/+/+/+/+/+/+/+ | +/+/+/+/+/+/+/+ | +/+/+/+/+/+/+/+ | +/+/+/+/+/+/+/+ | +/+/+/+/+/+/+/+ |
| Tp34 lipoprotein (TPAMA_0971) | + | + | + | + | - | - | - | + | + | + |
| TmpA (TPAMA_0768) | - | - | + | + | - | - | - | - | + | + |
| TmpB (TPAMA_0769) | - | - | + | + | - | - | - | - | + | + |
| TmpC (TPAMA_0319) | + | + | - | - | + | + | - | + | + | + |
| Haemolysin III | + | + | + | + | + | + | - | + | + | + |

Includes virulence factors present in more than one species. Virulence factors restricted to *T. denticola* or only the syphilis pathogens are reported in complete table in supplementary Table 1. *Treponema denticola* virulence factors surveyed were included on the basis of previous descriptions and comprised 13 genes. *Treponema pallidum* virulence factors included 31 genes as previously defined and recently reported Pallilysin (Tp0751) and associated protein Tp0750 .

**References:**

1. Svartström O, Mushtaq M, Pringle M, Segerman B. Genome-wide relatedness of Treponema pedis, from gingiva and necrotic skin lesions of pigs, with the human oral pathogen Treponema denticola. PLoS One. 2013;8(8):e71281.

2. Pětrošová H, Zobaníková M, Čejková D, Mikalová L, Pospíšilová P, Strouhal M, et al. Whole genome sequence of Treponema pallidum ssp. pallidum, strain Mexico A, suggests recombination between yaws and syphilis strains. PLoS Negl Trop Dis. 2012;6(9):e1832.

3. Houston S, Taylor JS, Denchev Y, Hof R, Zuerner RL, Cameron CE. Conservation of the Host-Interacting Proteins Tp0750 and Pallilysin among Treponemes and Restriction of Proteolytic Capacity to Treponema pallidum. Infect Immun. 2015;83(11):4204-16.
