# Supplementary material for: Dissecting the molecular diversity and commonality of bovine and human treponemes identifies key survival and adhesion mechanisms
Source: PLoS Pathog. 2021 Mar 29;17(3):e1009464. doi: 10.1371/journal.ppat.1009464 (PMC8049484; doi:10.1371/journal.ppat.1009464)
Supplement: S3 Table — (DOC) [file ppat.1009464.s003.doc]

**S3 Table. Identified** disease associated determinants differentiating bovine pathogenic and commensal treponemes

|  | ***Treponema medium* ATCC 700293^T^** | ***Treponema medium***  **T19**  **DSM 18689** | ***Treponema phagedenis***  **Reiter** | ***Treponema phagedenis***  **T320A**  **DSM 18690** | ***Treponema pedis* T3552B^T^**  **DSM 18691** | ***Treponema pedis***  **A4** | ***Treponema ruminis***  **DSM 103462^T^** | ***Treponema denticola***  **ATCC 35405** | ***Treponema pallidum*** ***subsp. pallidum Nichols*** | ***T. paraluiscuniculi***  ***Cuniculi A*** |
| --- | --- | --- | --- | --- | --- | --- | --- | --- | --- | --- |
| Host | human | bovine | Human | bovine | Bovine | porcine | bovine | human | human | rabbit |
| M18 aminopeptidase (DNPEP) (C5O78_01505) *(CYT)^1^* | + *(E)* | + *(E)* | + *(E)* | + *(E)* | + *(E)* | + *(E)* | - | + | - | - |
| Dipeptidase D (PepD) (C5O78_07270) *(CYT)* | + *(E)* | + *(E)* | + *(E)* | + *(E)* | + *(E)* | + *(E)* | - | + | - | - |
| Oligoendopeptidase F (PepF) (C5O78_13740) *(CYT)* | + *(E)* | + *(E)* | + *(E)* | + *(E)* | + *(E)* | + *(E)* | - | + | - | - |
| Signal peptidase II (LspA) (C5O78_08010) *(IM)* | + *(E)* | + *(E)* | + *(E)* | + *(E)* # | + *(E)* | + *(E)* | - | + | + | + |
| ATP dependent 6-phosphofructokinase (PfkA) (C5O78_12725) *(CYT)* | + *(E)* | + *(E)* | + *(E)* | + *(E)* | + *(E)* | + *(E)* | - | + | + | + |
| pyruvate kinase (PK) (C5O78_13165) *(CYT)* | + *(E)* | + *(E)* | + *(E)* | + *(E)* | + *(E)* | + *(E)* | - | + | - | - |
| Ferritin (FtnA) (C5O78_10015) *(CYT)* | + *(E)* | + *(E)* | + *(E)* | + *(E)* | + *(E)* | + *(E)* | - | + | - | - |
| UDP-N-acetyl-D-glucosamine dehydrogenase (WbpA/WbpO) (C5O78_00350) *(CYT)* | + *(E)* | + *(E)* | - | + *(E)* | + *(E)* | + *(E)* | - | - | - | - |
| UDP-N-acetyl-2-amino-2-deoxyglucuronate dehydrogenase (WbpB/WlbA) (C5O78_00440) *(CYT)* | + *(E)* | + *(E)* | - | + *(E)* | + *(E)* | + *(E)* | - | - | - | - |
| UDP-2-acetamido-2-deoxy-ribo-hexuluronate aminotransferase (WbpE/WlbC) (C5O78_00420) *(CYT)* | + *(E)* | + *(E)* | - | + *(E)* | + *(E)* | + *(E)* | - | - | - | - |
| UDP-2-acetamido-3-amino-2,3-dideoxy-glucuronate N-acetyltransferase (WbpD/WlbB) (C5O78_00405) *(CYT)* | + *(E)* | + *(E)* | - | + *(E)* | + *(E)* | + | - | - | - | - |
| UDP-GlcNAc3NAcA epimerase (WbpI /WlbD) (C5O78_10860) *(CYT)* | + *(E)* | + *(E)* | - | + *(E)* | + *(E)* | + *(E)* | - | - | - | - |
| Alpha-1,3-rhamnosyltransferase (WbdB) (C5O78_02140) *(CYT)* | + *(E)* | + *(E)* | - | + *(E)* | + *(E)* | + *(E)* | - | + | - | - |
| flhB2; flagellar biosynthesis protein: (C5O78_10975) *(IM)* | + | + | + | + | + | + | - | + | - | - |
| methyl-galactoside transport system ATP-binding protein (mglA) (C5O78_01835) *(IM)* | + *(E)* | + *(E)* | + *(E)* | + *(E)* | + *(E)* | + *(E)* | - | + | + | + |
| methyl-galactoside transport system substrate-binding protein (MglB) (C5O78_01830) (*PE*) | + *(E)* | + *(E)* | + *(E)* | + *(E)* | + *(E)* | + *(E)* | - | + | + | + |
| zinc transport system permease protein (ZnuB) (C5O78_05910) *(IM)* | + | + | + | + | + | + | - | + | - | - |
| putative ABC transport system ATP-binding (ABC.CD.A) (C5O78_03990) *(IM)* | + *(E)* | + *(E)* | + *(E)* | + *(E)* | + *(E)* | + *(E)* | - | + | - | - |
| ABC-2 type transport system permease protein: (ABC-2.P) (C5O78_12955) *(IM)* | + | + | + | + | + | + *(E)* | - | + | - | - |
| phosphocarrier protein (HPr) (C5O78_00635) *(CYT)* | + | + *(E)* # | + | + | + | + *(E)* # | - | + | - | - |
| magnesium transporter (MgtE) (C5O78_12755) *(IM)* | + *(E)* | + *(E)* | + *(E)* | + *(E)* | + *(E)* | + *(E)* | - | + | + | + |
| oligogalacturonide transporter (TogT) (C5O78_03835) *(IM)* | + *(E)* | + *(E)* | + | + *(E)* | + *(E)* | + *(E)* | - | + | + | + |
| C4-dicarboxylate transporter, DctM subunit (DctM) (C5O78_00075) *(IM)* | + | + *(E)* | + | + | + | + *(E)* | - | + | - | - |
| PPIA; peptidyl-prolyl cis-trans isomerase A (cyclophilin A) (C5O78_00570) *(CYT)* | + *(E)* | + *(E)* | + *(E)* | + *(E)* | + *(E)* | + *(E)* | - | + | - | - |
| chaperonin GroEL (groEL) (C5O78_01775) *(CYT)* | + *(E)* | + *(E)* | + *(E)* | + *(E)* | + *(E)* | + *(E)* | - | + | + | + |
| IMP dehydrogenase (IMPDH) (C5O78_11605) *(CYT)* | + *(E)* | + *(E)* | + *(E)* | + *(E)* | + *(E)* | + *(E)* | - | + | - | - |
| 2,3-bisphosphoglycerate-dependent phosphoglycerate mutase (PGAM) (C5O78_07690) *(CYT)* | + *(E)* | + *(E)* | + *(E)* | + *(E)* | + *(E)* | + *(E)* | - | + | + | + |

**^1^**^.^Predicted location of proteins are listed after each molecule in parentheses. IM =Inner membrane, CYT=Cytoplasm, PE=Periplasm, none localised to outer membrane. Expression of corresponding protein identified using proteomics is denoted by *E* in parentheses. All putative OMPs detected in a minimum of two peptide identifications except # which only had one.
