# Supplementary material for: Dissecting the molecular diversity and commonality of bovine and human treponemes identifies key survival and adhesion mechanisms
Source: PLoS Pathog. 2021 Mar 29;17(3):e1009464. doi: 10.1371/journal.ppat.1009464 (PMC8049484; doi:10.1371/journal.ppat.1009464)
Supplement: S4 Table — (DOC) [file ppat.1009464.s004.doc]

**S4 Table 4. Unique genes with putative functions attributed to bovine and human *Treponema phagedenis* and their presence within relevant human and bovine strains.**

| Location | ***Description*** | ***Locus tag*** | ***Treponema phagedenis***  **Strain Reiter ^3^** | ***Treponema phagedenis* T320A**  **DSM 18690 ^3^** | ***Treponema phagedenis* bovine 4A USA** | ***Treponema phagedenis* human F0421 USA** | ***Treponema phagedenis* bovine V1 Sweden** | ***Presence in 9 UK DD trep and 2 human as determined by PCR^4^*** |
| --- | --- | --- | --- | --- | --- | --- | --- | --- |
| Host |  |  | human | Bovine | Bovine | human | bovine | Bovine/human |
| **Phosphate**  **Utilisation**  **Cluster** |  | | | | | | | |
|  | Mcp: Methyl accepting chemotaxis protein | C5078-02335 | - | + | + | - | + | **9/0, P=0.0182*** |
|  | GHKL domain containing protein | C5078-02340 | - | + (*E*) | + | - | + |  |
|  | PhoB: DNA binding response regulator | C5078-02340 | - | + (*E*) | + | - | + | **9/0, P=0.0182*** |
|  | PhoU: Phosphate transport system regulatory protein | C5078-02355 | - | + (*E*) | + | - | + |  |
|  | PstB: Phosphate transport system ATP-binding protein | C5078-02355 | - | + (*E*) | + | - | + | **9/0, P=0.0182*** |
|  | PstA: Phosphate ABC transporter permease | C5078-02360 | - | + | + | - | + |  |
|  | PstC: Phosphate ABC transporter permease subunit C | C5078-02365 | - | + | + | - | + | **9/0, P=0.0182*** |
|  | PstS: Phosphate binding protein | C5078-02370 | - | + | + | - | + |  |
|  | Transcriptional regulator | C5078-02385 | - | + | + | - | + | **9/0, P=0.0182*** |
|  | GTP-binding domain protein | C5078-02400 | - | + | + | - | + |  |
|  |  |  |  |  |  |  |  |  |
| **Citric Acid /Secretion System Cluster** | CitD: citrate lyase acyl carrier protein | C5078-13930 | - | + | + | - | + | **9/0, P=0.0182*** |
|  | CitE: citrate lyase subunit beta | C5078-13935 | - | + | + | - | + | **9/0, P=0.0182*** |
|  | CitF: citrate lyase subunit alpha | C5078-13940 | - | + | + | - | + | **9/0, P=0.0182*** |
|  | Citrate-sodium symporter | C5078-13945 | - | + | + | - | + | ND |
|  | Type III endonuclease | C5078-13955 | - | + | + | - | + | ND |
|  | Site specific DNA methyl transferase | C5O78_13960 | - | + (*E*) | + | - | + | ND |
|  | ATP Binding Protein | C5O78_13965 | - | + | + | - | + | ND |
|  | Fibronectin binding protein | C5O78_13970 | - | + | + | - | + | ND |
|  | ABC transporter | C5O78_13975 | - | + | + | - | + | ND |
|  | EcfT | C5O78_13980 | - | + | + | - | + | ND |
|  | TetR/AcrR transcriptional regulator | C5O78_13995 | - | + | + | - | + | ND |
|  | Cell filamentation protein Fic | C5O78_14000 | - | + | + | - | + | ND |
|  | Class 1 SAM dependent methyl transferase | C5O78_14005 | - | + | + | - | + | **9/0, P=0.0182*** |
|  | N-acetyl transferase | C5O78_14025 | - | + | + | - | + | ND |
|  | Cell filamentation protein Fic | C5O78_14030 | - | + | + | - | + | **9/0, P=0.0182*** |
|  | Cell filamentation protein Fic | C5O78_14040 | - | + | + | - | + | ND |
|  | Methyltransferase | C5O78_14055 | - | + | + | - | + | ND |
|  | TraG/TraD/VirD4 bacterial conjugation protein | C5O78_14060 | - | + | + | - | + | **9/0, P=0.0182*** |
|  | DNA Topoisomerase III | C5O78_14075 | - | + | + | - | + | ND |
|  | AbrB/MazE/SpoVT family DNA binding protein containing protein | C5O78_14080 | - | + | + | - | + | ND |
|  | Class 1 SAM dependent methyl transferase | C5O78_14090 | - | + | + | - | + | ND |
|  | Conjugal transfer protein | C5O78_14095 | - | + | + | - | + | ND |
|  | XRE transcriptional regulator | C5O78_14100 | - | + | + | - | + | ND |
|  | Site specific DNA methyl transferase | C5O78_14105 | - | + | + | - | + | **9/0, P=0.0182*** |
|  | DNA cytosine methyl transferase | C5O78-14130 | - | + | + | - | + | ND |
| **YcaO Cluster^1^** | YcaO-like protein | C5O78-09450 | - | + | - | - | + | ND |
|  | Xenobiotic-transporting ATPase | C5O78-09455 | - | + | - | - | + | ND |
|  | ABC Transporter | C5O78-09460 | - | + | - | - | + | ND |
|  | ABC Transporter Permease | C5O78-09465 | - | + | - | - | + | ND |
|  | Iron complex ABC Transporter | C5O78-09470 | - | + | - | - | + | ND |
| **LPS synthesis/ Oxidative** | Glycosylyltranserase (WBuB) | C5O78-00345 | - | + | + | - | + | ND |
| **Stress Cluster** | UDP-N-acetyl-D-glucosamine dehydrogenase (WbpA/WbpO) | C5O78-00350 | - | + | + | - | + | ND |
|  | UDP-N-Acetylglucosamine 2-epimerase (non-hydrolysing) (WecB) | C5O78-00355 | - | + | + | - | + | ND |
|  | Glycosyltransferase | C5O78_00360 | - | + | + | - | + | ND |
|  | Lipopolysaccharide/Colanic Acid exporter (WzxC) | C5O78_00380 | - | + (*E*) | + | - | + | ND |
|  | NADP Oxidoreductase | C5O78_00385 | - | + (*E*) | + | - | + | **9/0, P=0.0182*** |
|  | ATP grasp domain containing protein | C5O78_00390 | - | + (*E*) | + | - | + | ND |
|  | dTDP-4-amino-4,6-dideoxygalactose transaminase (WecE) | C5O78_00395 | - | + (*E*) | + | - | + | ND |
|  | glucose-1-phosphate thymidylyltransferase (RfbA) | C5O78_00400 | - | + (*E*) | + | - | + | ND |
|  | UDP-2-acetamido-3-amino-2,3-dideoxy-glucuronate N-acetyltransferase (WbpD/WlbB) | C5O78_00405 | - | + (*E*) | + | - | + | ND |
|  | Nucleotidyltransferase | C5O78_00410 | - | + | + | - | + | ND |
|  | UDP-2-acetamido-2-deoxy-ribo-hexuluronate aminotransferase (WbpE/WlbC) | C5O78_00420 | - | + (*E*) | + | - | + | **9/0, P=0.0182*** |
|  | UDP-N-acetyl-2-amino-2-deoxyglucuronate dehydrogenase (WbpB/WlbA) | C5O78_00440 | - | + (*E*) | + | - | + | **9/0, P=0.0182*** |
|  | Aminotransferase class V fold PLP dependent enzyme | DWQ65_02920 | + (*E*) | - | - | + | - | ND |
| **Sugar transporter cluster** | Carbohydrate ABC transporter permease | DWQ65_02925 | + | - | - | + | - | ND |
|  | Sugar ABC transporter permease | DWQ65_02930 | + | - | - | + | - | ND |
|  | Sugar ABC transporter permease substrate binding protein | DWQ65_02935 | + (*E*) | - | - | + | - | ND |
| VpsA,PrrA, VpsB Cluster^2^ | Methyl accepting chemotaxis protein | C5O78_02860 | - | + | - | - | + | ND |
|  | VpsA | C5O78_02885 | - | + (*E*) | + | - | + | ND |
|  | PrrA | - | - | - | + | - | + | ND |
|  | VpsB | C5O78_02905 | - | + (*E*) | + | - | + | ND |
| Single gene | Uridine kinase | C5O78_00495 | - | + (*E*) | + | + | + | ND |
| Single gene | Single stranded DNA binding protein | DWQ65_02235 | + | - | - | + | - | ND |

Unique genes identified through pan genome analysis with BPGA program and then validated using mauve alignment. Only those genes with annotated function are listed. ^1^ Previously reported ^2^previously reported . ^3^Expression of corresponding protein identified using proteomics is denoted by *E* in parentheses. ^4^PCR presence of genes was identified on a single gene basis for all loci except for the phosphate utilisation gene cluster which were identified using PCR targeting two adjacent genetic loci simultaneously. The relationship between bovine and human strains and presence of genes was investigated using Fisher’s exact test (two-tailed).

**References:**

1. Mushtaq M, Bongcam-Rudloff E, Loftsdottir H, Pringle M, Segerman B, Zuerner R, et al. Genetic analysis of a Treponema phagedenis locus encoding antigenic lipoproteins with potential for antigenic variation. Vet Microbiol. 2016;189:91-8.

2. Rosander A, Guss B, Frykberg L, Bjorkman C, Naslund K, Pringle M. Identification of immunogenic proteins in Treponema phagedenis-like strain V1 from digital dermatitis lesions by phage display. Vet Microbiol. 2011;153(3-4):315-22.
