# Supplementary material for: Dissecting the molecular diversity and commonality of bovine and human treponemes identifies key survival and adhesion mechanisms
Source: PLoS Pathog. 2021 Mar 29;17(3):e1009464. doi: 10.1371/journal.ppat.1009464 (PMC8049484; doi:10.1371/journal.ppat.1009464)
Supplement: S5 Table — (DOC) [file ppat.1009464.s005.doc]

**S5 Table. Primers used to confirm the distribution of select genes within gene clusters across *Treponema phagedenis* strains**.

| **Oligoname** | **Primer** | **Sequence** | **Tm** | **Product size** |
| --- | --- | --- | --- | --- |
| Cell filamentation protein  (C5O78_14030) | FicForward | atatataaaagagtccgagccgaaa | 60.6 | 1105 |
|  | FicReverse | aaatctgtatttcccttttccatgt | 60.3 |  |
| Class 1 SAM dependent methyl transferase  (C5O78_1400) | SAMForward | gtgccgaaaaacatgttgtacttat | 60.5 | 665 |
|  | SAMReverse | acgttttcatagcccatatctttta | 59.4 |  |
| Site specific DNA methyl transferase (C5O78_14105) | SSDNAForward | agagctgaaatgcttgaatacctaa | 59.8 | 1249 |
|  | SSDNAReverse | gcttcaaaacctttgattttactga | 60.1 |  |
| TraG/TraD/VirD4 bacterial conjugation protein (C5O78_14060) | VirD4Forward | tctatgaaatacaatccctttgcat | 60.1 | 447 |
|  | VirD4Reverse | aagctcaagttcatcttcactcatt | 59.9 |  |
| CitE: citrate lyase subunit beta (C5078_13935) | CitE forward | gattacgcagaacaatgatgttttt | 60.4 | 735 |
|  | CitE reverse | atttttccgtctaccgcaattact | 61.3 |  |
| CitF: citrate lyase subunit alpha (C5078_13940) | CitF forward | aaaaatgcggtaggaagaaata | 57.1 | 1414 |
|  | CitF reverse | attcttcgatactgcgtatgggta | 61 |  |
| CitD: citrate lyase acyl carrier protein (C5078-13930) | CitD forward | agctaaaaacaactggggttgc | 61.7 | 274 |
|  | CitD reverse | cattgataatcggttctttcagc | 60 |  |
| UDP-2-acetamido-2-deoxy-ribo-hexuluronate aminotransferase (WbpE/WlbC) (C5O78_00420) | DegT F | cgtgaatatcaaaactataagcagga | 60 | 1081 |
|  | DegT R | gtagctgacttcgtcttcggttaat | 58.8 |  |
| UDP-N-acetyl-2-amino-2-deoxyglucuronate dehydrogenase (WbpB/WlbA) (C5O78_00440) | MocA F | taattgccgtatgtgatcctatttt | 60 | 1072 |
|  | MocA R | ctaaggttgaaaactcacaaggaag | 59.8 |  |
| NADP Oxidoreductase (C5O78_00385) | NADPoxidoreduct F | tggtttggttggtcaatacttagtt | 60.1 | 918 |
|  | NADPoxidoreduct R | tttatatcgcgcaacatttctttat | 60.2 |  |
| Mcp: Methyl accepting chemotaxis protein (C5078-02335)/ GHKL domain containing protein (C5078-02340) | MeGHKLFwd | ttaaaattgtaaatccgtcttctgc | 56.4 | 433 |
|  | MeGHKLRev | tgaactggaagctattatccttgac | 59.7 |  |
| PhoB: DNA binding response regulator (C5078-02340) / PhoU: Phosphate transport system regulatory protein (C5078-02355) | DNArrPhoUFwd | ttgacaatatttgcacatcaatagc | 56.4 | 826 |
|  | DNArrPhoURev | aaaaagagatgatgagattgacagg | 58.1 |  |
| PstB: Phosphate transport system ATP-binding protein (C5078-02355) / PstA: Phosphate ABC transporter permease (C5078-2360) | PstBPstAFwd | attagctcttcaatttttgcagttg | 56.4 | 1358 |
|  | PstBPstARev | cggaaaaacgcttatttattttaca | 54.8 |  |
| PstC: Phosphate ABC transporter permease subunit C (C5078-02365) / PstS: Phosphate binding protein (C5078-02370) | PstCPBSFwd | gaataaaaatctgctcacccttgta | 58.1 | 851 |
|  | PstCPBSRev | aagacctccctatccttgttgtaaa | 59.7 |  |
| Transcriptional regulator (C5078-02385) | TranRFwd | aatattgcagctggacgaagtaata | 58.1 | 573 |
|  | TranRRev | agcctataacgtaccaaactcctct | 61.3 |  |
