# Supplementary material for: Dissecting the molecular diversity and commonality of bovine and human treponemes identifies key survival and adhesion mechanisms
Source: PLoS Pathog. 2021 Mar 29;17(3):e1009464. doi: 10.1371/journal.ppat.1009464 (PMC8049484; doi:10.1371/journal.ppat.1009464)
Supplement: S6 Table — (DOC) [file ppat.1009464.s006.doc]

**S6 Table: Primers used for putative OMP gene amplification to enable gene cloning and expression**.

| Locus tag | *Treponema* Phylogroup | Sense primer (5’-3’) | Antisense primer (5’-3’) | Amplicon size (kb) | Annealing temperature (^o^C) |
| --- | --- | --- | --- | --- | --- |
| C5N99_04710 | *T. medium* | CACCCCGGTAGATTTGTTCGATC | CTAATACGCAAACTCTGCG | 1.8 | 59.2 |
| C5N99_04715 | *T. medium* | CACCGATACGCCTTATGCCTTATTTG | TTACCGAATCGTATATTTCACAG | 1.6 | 62.9 |
| C5N99_04785 | *T. medium* | CACCGCTGCCGAAACTCAAGAAC | TTAGAAGTGATATCGGGCG | 0.6 | 60.7 |
| C5N99_05000 | *T. medium* | CACCGTAAAATCTTTTGATACCGCTG | TTACCGCCCTAAGCGGA | 1.0 | 59.7 |
| C5N99_05295 | *T. medium* | CACCACGGAAACAGGGCTTGCTC | TTACCGCTTTTCAAACAGCAG | 1.4 | 64.4 |
| C5N99_06860 | *T. medium* | CACCCTGCCTGCGTACTTTGTA | TCAATATGTGTCCTCCATTTGC | 0.6 | 58.1 |
| C5N99_06875 | *T. medium* | CACCGATAATTGGTATGAAAATAAACCG | TTATAGGTTAGGTACATTAAACG | 2.4 | 57.2 |
| C5N99_06910 | *T. medium* | CACCAACGTAAAGATGAGCCTTGATG | TTAAAAGACGAATTGAACACCG | 0.7 | 65.4 |
| C5N99_03545 | *T. medium* | CACCGACGCGGTGCAAACTGAAAC | TTAGAACTTGCCGCCGCCGA | 1.5 | 68.2 |
| C5N99_04545 | *T. medium* | CACCTAGATACTAGTACCGATTCTG | CTAGAAACCGATACCTACGC | 1.6 | 50.9 |
| C5N99_10335 | *T. medium* | CACCGATGGGGTCGATTTTTCG | CTACAGCTTAAAAGCGATCC | 0.7 | 58.7 |
| C5N99_10205 | *T. medium* | CACCCAGCAGACTGCTGATTCTG | CCACCAACTCTTTAGAATTC | 1.2 | 55.9 |
| C5N99_00085 | *T. medium* | CACCGAGAATACTCGCGATGGA | TTACCAGCTGTCTTTCATATCGTCA | 0.7 | 59.9 |
| C5N99_03350 | *T. medium* | CACCCAAACTAATCTGCAGCCTATTGC | CCATTTTTACCACGAAAGCG | 0.7 | 64.0 |
| C5N99_02965 | *T. medium* | CACCCAGGAAGAAGGAGCAGAGG | AGAGATACCCATTAGTTGTTG | 0.9 | 55.4 |
| C5O78_02150 | *T. phagedenis* | CACCGCGACTGATATTTTTGATCC | TTAGTATGCAAACTCCGCAC | 1.7 | 59.1 |
| C5O78_02155 | *T. phagedenis* | CACCCAAGAAAATATGGCAAAAATAATCC | TCTTATCGTATACTATAATTTATCG | 1.6 | 56.1 |
| C5O78_07955 | *T. phagedenis* | CACCTCCGAGCAGGAAACTACTG | TCAAAAGTGATATCGTGCTC | 0.7 | 58.4 |
| C5O78_03185 | *T. phagedenis* | CACCGTAAAATCTTTTGATACCGCTG | TTACCGCCCTAAGCGGA | 1.0 | 59.7 |
| C5O78_04000 | *T. phagedenis* | CACCGCGGAAGAAAATTTATATGATGC | TTATTTGTCTCCTTGTACCGA | 1.5 | 62.2 |
| C5O78_01225 | *T. phagedenis* | CACCTATGTTGTGCAATATAAAGAGC | TTAGTAGGTTGTGTCCATATTCA | 0.6 | 59.7 |
| C5O78_01240 | *T. phagedenis* | CACCGTTGCGGATAACTGGTATG | CTCCTTTATATCATAAATTAGGTG | 2.5 | 55.1 |
| C5O78_10020 | *T. phagedenis* | CACCAAGCAGTATGGCATTAATG | TTAAAAATGAAAGCGTAACCCG | 0.6 | 55.8 |
| C5O78_01255 | *T. phagedenis* | CACCGAACATGGTTAAACGAAGAA | TTAGAGCATAGAGCGGAACGGAATATT | 1.1 | 60.7 |
| C5O78_08250 | *T. phagedenis* | CACCGAAATTGAGTTATCAAAATATC | TTAAAAACCTAAAGCTAATCCCAG | 1.3 | 55.3 |
| C5O78_05585 | *T. phagedenis* | CACCGCGGAAGTTTCAATTTCTGT | CTTAGAATTTTACCGATAAGCC | 0.7 | 58.1 |
| C5O78_05635 | *T. phagedenis* | CACCACAATCGAGCAGCAAAATTCTC | TTAGAATTCCCATCCGACG | 1.2 | 62.8 |
| C5O78_09920 | *T. phagedenis* | CACCCAAGAAGAACAAAGCGCAAG | TTACCAGTTATCAGCCATGTCA | 0.8 | 62.4 |
| C5O78_07735 | *T. phagedenis* | CACCGCTTTGCAGCCGATTGCT | TTACCAAGCTAGAAGTTTTTTC | 1.1 | 56.6 |
| C5O78_04920 | *T. phagedenis* | CACCGAAGAGAGTAGTCAATCAACG | TTAGTTATGGTTGAATGTCATG | 1.0 | 59.2 |
| DYQ05_09320 | *T. pedis* | CACCGCCGTAGATGTTTTAGACC | GCTTAGTACGTAATTTTTATTGC | 1.7 | 56.0 |
| DYQ05_09315 | *T. pedis* | CACCCTTTATGTAATTCTTATAC | TTACCGCAGAGAATACATTATAT | 1.6 | 50.9 |
| DYQ05_01630 | *T. pedis* | CACCGCGGAAGGCTGGTATAACG | ACTCCTCATTTATTAAATATTAGG | 2.4 | 54.0 |
| DYQ05_13425 | *T. pedis* | CACCTTAAGCGATATTTCAGGCGATG | TTACAGCTTCCATGCAATACC | 0.8 | 64.9 |
| DYQ05_10935 | *T. pedis* | CACCGCACAAAGCGATTTACAGG | TTACCAAGAAAGAATCTTGTCC | 1.0 | 59.2 |
| DYQ05_07395 | *T. pedis* | CACCGAAGAAGGAGGGAAGGAAG | TTAAAAATGATACCTTGCGGC | 0.6 | 59.9 |
| DYQ05_12540 | *T. pedis* | CACCGCGGAAAACGGAGTTTC | TTAAAATTCCCAACCCAAAGAAAG | 1.2 | 61.3 |
| DYQ05_12210 | *T. pedis* | CACCCAGGAAGAAGCAAATACC | TTACCAGTTATCGTTCATATCG | 0.8 | 55.3 |
| DYQ05_07390 | *T. pedis* | CACCTGTGAAGAAGAAAAAAAATCCG | CTAAAAACTAAACGACACG | 0.9 | 53.2 |
| DYQ05_01950 | *T. pedis* | CACCGAAAATATTTATAATAACCTTCTCG | CCATTATTTACCTCCGGTTTC | 1.5 | 58.8 |
| DYQ05_01600 | *T. pedis* | CACCTCGATATTCCCTTATTTTGTGG | TTAATATGTTTTTTCGTCCATATCC | 0.6 | 64.0 |
| DYQ05_06810 | *T. pedis* | CACCGCAAAGACTATCGGTCTTAATTTG | TTAAAAATAAACTCTTAAACCCGC | 0.6 | 63.6 |
| DYQ05_09195 | *T. pedis* | CACCCAGGAAAATAAAGAGAATCTTG | TTAAAAATTACCGCCGCC | 1.0 | 56.8 |
| DYQ05_12175 | *T. pedis* | CACCTCTCAAGAAAGCGTTTCTTC | TTAATACCCCACAACCACC | 1.4 | 59.3 |
| DYQ05_07280 | *T. pedis* | CACCGTTATAAACGGAATTTATACCG | TTACTCTCCTAAACGTATCATC | 1.0 | 57.8 |
