# Supplementary material for: Dissecting the molecular diversity and commonality of bovine and human treponemes identifies key survival and adhesion mechanisms
Source: PLoS Pathog. 2021 Mar 29;17(3):e1009464. doi: 10.1371/journal.ppat.1009464 (PMC8049484; doi:10.1371/journal.ppat.1009464)
Supplement: S7 Table — (DOC) [file ppat.1009464.s007.doc]

**S7 Table: Secondary structure and fold recognition based on circular dichroism spectrometry of treponeme putative OMPs.**

|  | Secondary structure determination and fold recognition^#^ | | | | | | | | | | | |  |  | |
| --- | --- | --- | --- | --- | --- | --- | --- | --- | --- | --- | --- | --- | --- | --- | --- |
| locus tag | | α-Helix | Antiparallel | Parallel | Turn | Others | Helix1 (regular) | Helix2 (distorted) | Anti1 (left-twisted) | Anti2 (relaxed) | Anti3 (right-twisted) | Predominant structure | Concentration (mg/ml) | Solubility (14 days, 4^o^C)* |  |
| C5N99_04710 | | *n.d.* | | | | | | | | | | |  |  |  |
| C5O78_02150 | | *n.d.* | | | | | | | | | | |  |  |  |
| DYQ05_09320 | | 6 | 34.3 | 1.4 | 13.8 | 44.6 | 3.8 | 2.2 | 4.2 | 17.1 | 13 | β-sheet | 1.0 | + |  |
| C5N99_04715 | | *n.d.* | | | | | | | | | | |  |  |  |
| C5O78_02155 | | 14.8 | 32 | 0 | 12.4 | 40.9 | 8 | 6.8 | 4.8 | 13 | 14.2 | β-sheet | 1.0 | + |  |
| DYQ05_09315 | | *n.d.* | | | | | | | | | | |  |  |  |
| C5N99_04785 | | 12.1 | 27.9 | 0.1 | 11.8 | 48.1 | 5.5 | 6.6 | 4.3 | 11.7 | 11.9 | β-sheet | 1.4 | + |  |
| C5O78_07955 | | *n.d.* | | | | | | | | | | |  |  |  |
| DYQ05_07395 | | 2.1 | 48 | 0 | 11.7 | 38.3 | 2.1 | 0 | 4.8 | 21.1 | 22.1 | β-sheet | 1.0 | + |  |
| C5N99_05295 | | *n.d.* | | | | | | | | | | |  |  |  |
| C5O78_04000 | | *n.d.* | | | | | | | | | | |  |  |  |
| DYQ05_01950 | | 1.2 | 36.9 | 0.8 | 15 | 46.1 | 0.8 | 0.4 | 3.8 | 15.3 | 17.9 | β-sheet | 0.5 | + |  |
| C5N99_06860 | | *n.d.* | | | | | | | | | | |  |  |  |
| C5O78_01225 | | 20.9 | 16.4 | 1.8 | 13.3 | 47.6 | 12 | 8.9 | 1.4 | 10.3 | 4.7 | α-helix | 1.0 | + |  |
| DYQ05_01600 | | 1.3 | 42.8 | 0 | 13.6 | 42.3 | 1.3 | 0 | 5.2 | 18.7 | 18.7 | β-sheet |  | + |  |
| C5N99_06910 | | 0 | 37.2 | 3.8 | 14.2 | 44.8 | 0 | 0 | 4.1 | 16.3 | 16.8 | β-sheet | 0.8 | + |  |
| C5O78_10020 | | *n.d.* | | | | | | | | | | |  |  |  |
| DYQ05_06810 | | 4.7 | 42.5 | 0 | 12.6 | 40.1 | 3.2 | 1.6 | 6.2 | 19.2 | 17.1 | β-sheet | 1.0 | + |  |
| C5N99_03545 | | *n.d.* | | | | | | | | | | |  |  |  |
| C5O78_01255 | | *n.d.* | | | | | | | | | | |  |  |  |
| DYQ05_09195 | | 9.6 | 23.4 | 2.3 | 15.1 | 49.6 | 4.2 | 5.4 | 0.3 | 11.4 | 11.7 | β-sheet | 1.0 | + |  |
| C5N99_10335 | | 10.4 | 33.1 | 6.2 | 11.2 | 39 | 4.1 | 6.3 | 6 | 10.9 | 16.2 | β-sheet | 4.6 | + |  |
| C5O78_05585 | | *n.d.* | | | | | | | | | | |  |  |  |
| DYQ05_13425 | | 5.9 | 38.6 | 1.2 | 12.7 | 41.6 | 3.3 | 2.6 | 4.6 | 16.7 | 17.3 | β-sheet | 1.0 | + |  |
| C5N99_02965 | | 12.6 | 29.2 | 3.8 | 13.6 | 40.8 | 5.1 | 7.5 | 2.9 | 13.9 | 12.4 | β-sheet | 2.3 | + |  |
| C5O78_05635 | | *n.d.* | | | | | | | | | | |  |  |  |
| DYQ05_12540 | | 10.2 | 25.3 | 0 | 15.2 | 49.3 | 4.7 | 5.5 | 0 | 11.2 | 14.1 | β-sheet | 1.0 | + |  |
| C5N99_10205 | | 1.4 | 48.3 | 0 | 13.1 | 37.2 | 0 | 1.4 | 0.2 | 18.4 | 29.7 | β-sheet | 0.5 | + |  |
| C5O78_04920 | | *n.d.* | | | | | | | | | | |  |  |  |
| DYQ05_07390 | | *n.d.* | | | | | | | | | | |  |  |  |

**# Secondary structure composition analysis performed by Bestsel**

*** Solubility in buffer (20 mM Tris HCl pH 7.9, 50 mM NaCl, 0.1% LDAO)**

**References:**

1. Micsonai A, Wien F, Bulyáki É, Kun J, Moussong É, Lee YH, et al. BeStSel: a web server for accurate protein secondary structure prediction and fold recognition from the circular dichroism spectra. Nucleic Acids Res. 2018;46(W1):W315-w22.
