# Supplementary material for: Dissecting the molecular diversity and commonality of bovine and human treponemes identifies key survival and adhesion mechanisms
Source: PLoS Pathog. 2021 Mar 29;17(3):e1009464. doi: 10.1371/journal.ppat.1009464 (PMC8049484; doi:10.1371/journal.ppat.1009464)
Supplement: S8 Table — (DOC) [file ppat.1009464.s008.doc]

**S8 Table. Relative optical density of four *T. phagedenis* strains post-exposure to distilled deionised water.**

| **Strain/Host** | **Mean 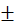 SEM*^a^*** |  |  | ***P* value^d^** |
| --- | --- | --- | --- | --- |
|  | **PBS OD_540_*^b^*** | **dH_2_O OD_540_** | **Relative OD_540_ (%)*^c^*** |  |
| **Reiter/Human** | 0.209 ± 0.003 | 0.129 ± 0.002 | 61.65±0.983 | - |
| **T320A /Bovine** | 0.229 ± 0.005 | 0.197 ± 0.007 | 85.93±1.822 | <0.0001**** |
| **DD1R /Bovine** | 0.271 ± 0.008 | 0.217 ± 0.011 | 79.89±2.667 | 0.0008*** |
| **T354B /Bovine** | 0.258 ± 0.012 | 0.233 ± 0.006 | 90.32±3.267 | <0.0001**** |

^a^ Values represent the means of 4 independent experiments

^b^ Turbidity (OD_540_) measured at 540nm after a 90-minute incubation at 37°C

^c^ Change in OD_540_ in distilled deionised H_2_O relative to PBS.

^d^ Turkey’s multiple comparison test result comparing each of the bovine strains with the human strain
