# Supplementary material for: Dissecting the molecular diversity and commonality of bovine and human treponemes identifies key survival and adhesion mechanisms
Source: PLoS Pathog. 2021 Mar 29;17(3):e1009464. doi: 10.1371/journal.ppat.1009464 (PMC8049484; doi:10.1371/journal.ppat.1009464)
Supplement: S9 Table — (DOC) [file ppat.1009464.s009.doc]

**S9 Table. Genome assembly characteristics of the assembled treponeme** genomes.

| Category^a^ | ***Treponema medium* ATCC 700293^T^** | ***Treponema medium***  **T19**  **DSM 18689** | ***Treponema phagedenis***  **Reiter** | ***Treponema phagedenis***  **T320A**  **DSM 18690** | ***Treponema pedis* T3552B^T^**  **DSM 18691** | ***Treponema ruminis***  **DSM 103462^T^** |
| --- | --- | --- | --- | --- | --- | --- |
| Host | human | bovine | human | bovine | Bovine | bovine |
| Number of raw reads | 3,881,158 | 4,239,942 | 4,658,044 | 4,350,662 | 3,069,010 | 4,614,304 |
| Number of trimmed reads | 3,871,755 | 4,227,347 | 4,642,413 | 4,334,193 | 3,029,634 | 4,594,190 |
| Mean assembly length | 13,182 | 4,050 | 18,603 | 9,135 | 5,854 | 36,419 |
| Q scores | 30.72 | 31.13 | 31.09 | 30.95 | 30.84 | 31.00 |
| Coverage (%) | 98.7 | 99.1 | 98.6 | 98.4 | 98.9 | 97.1 |
| Genbank Accession No^c^ | **CP031393** | **CP027017** | **CP031394** | **CP027018** | **CP045760** | **CP031518** |
