# Supplementary figures and images for: Dissecting the molecular diversity and commonality of bovine and human treponemes identifies key survival and adhesion mechanisms
Source: PLoS Pathog. 2021 Mar 29;17(3):e1009464. doi: 10.1371/journal.ppat.1009464 (PMC8049484; doi:10.1371/journal.ppat.1009464)

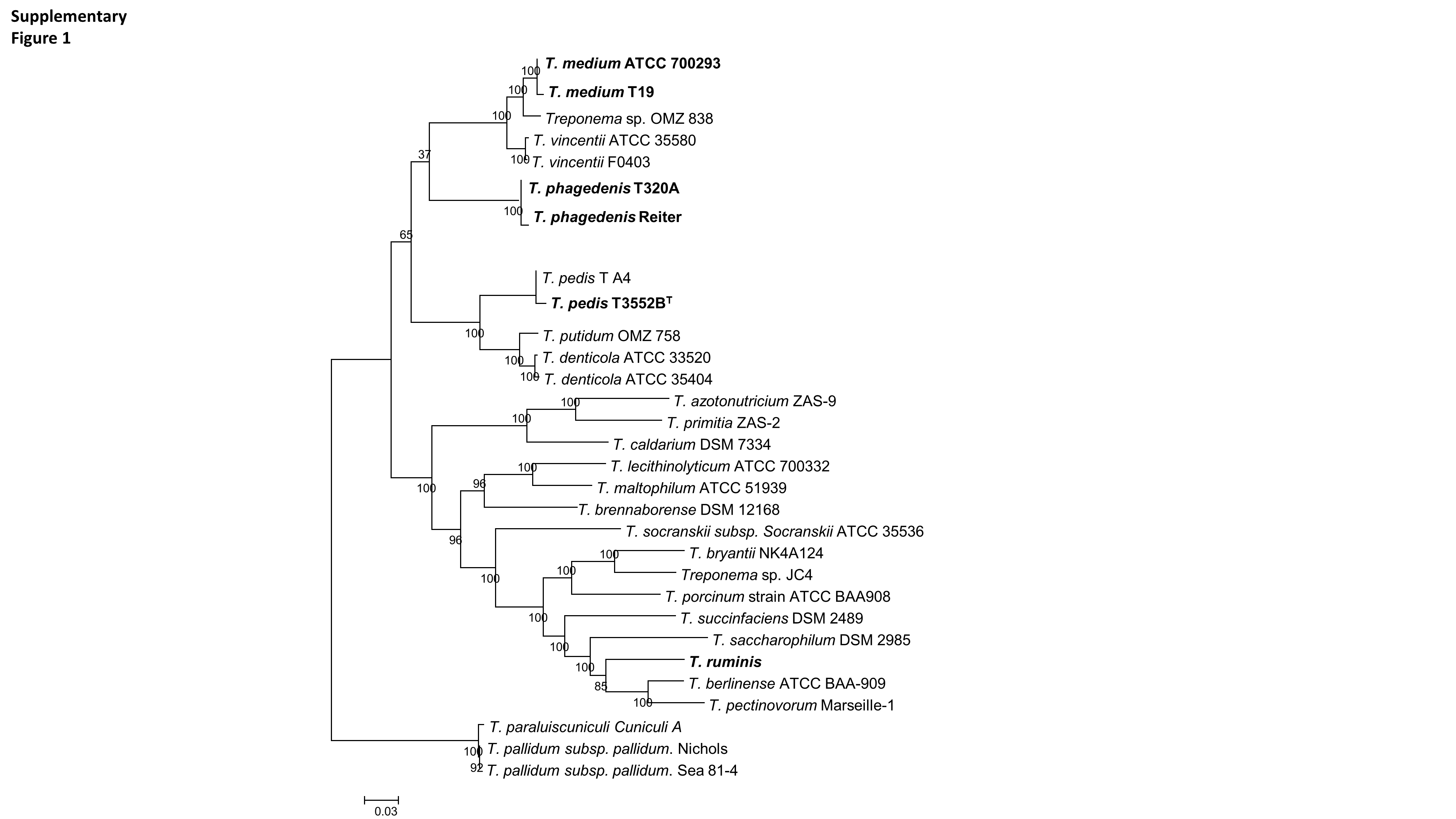

Supplement: S1 Fig — Ribosomal gene sequences were obtained, aligned and concatenated and a maximum likelihood tree generated using Mega 7.0 (88), the general time reversible model, as determined by Topali [89] with bootstrapping using 100,000 iterations. (TIF) [file ppat.1009464.s010.TIF]

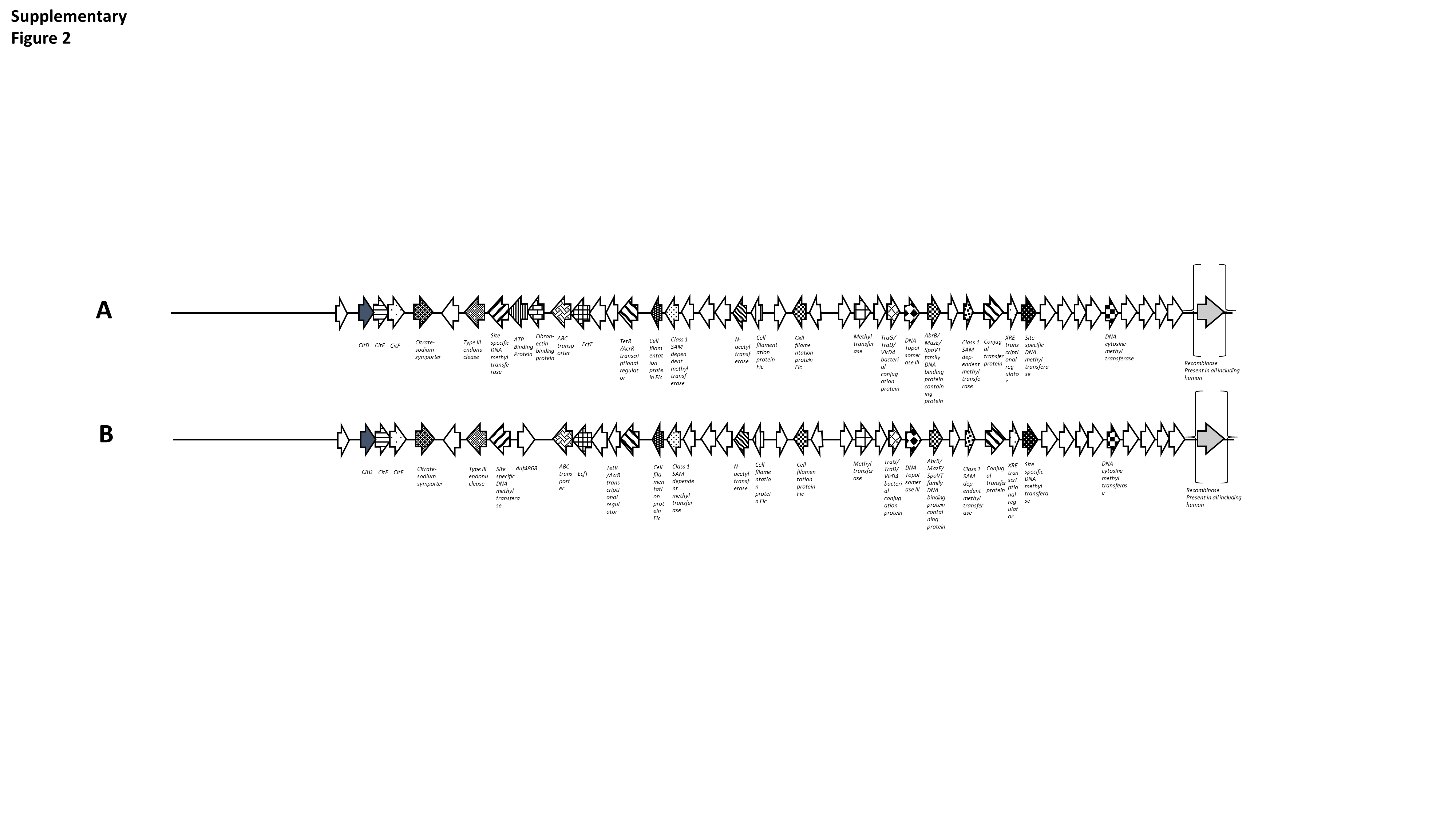

Supplement: S2 Fig — The bovine T. phagedenis unique gene cluster containing Type IV secretion system components, a citrate utilisation cluster cell filamentation proteins with either a fibronectin binding protein (A: UK and Sweden bovine strains) or a duf4868 protein (B: USA bovine strain). All ORFs present in UK, USA and Swedish T. phagedenis genomes and absent for human genomes except USA bovine strain has no ATP binding protein and fibronectin binding protein and instead has a duf4868 containing protein. (TIF) [file ppat.1009464.s011.TIF]
